# RWOA: A novel enhanced whale optimization algorithm with multi-strategy for numerical optimization and engineering design problems

**DOI:** 10.1371/journal.pone.0320913

**Published:** 2025-04-28

**Authors:** Junhao Wei, Yanzhao Gu, Baili Lu, Ngai Cheong

**Affiliations:** 1 Faculty of Applied Sciences, Macao Polytechnic University, Macao, China; 2 College of Animal Science and Technology, Zhongkai University of Agriculture and Engineering, Guangzhou, China; Griffith University, AUSTRALIA

## Abstract

Whale Optimization Algorithm (WOA) is a biologically inspired metaheuristic algorithm with a simple structure and ease of implementation. However, WOA suffers from issues such as slow convergence speed, low convergence accuracy, reduced population diversity in the later stages of iteration, and an imbalance between exploration and exploitation. To address these drawbacks, this paper proposed an enhanced Whale Optimization Algorithm (RWOA). RWOA utilized Good Nodes Set method to generate evenly distributed whale individuals and incorporated Hybrid Collaborative Exploration strategy, Spiral Encircling Prey strategy, and an Enhanced Spiral Updating strategy integrated with Levy flight. Additionally, an Enhanced Cauchy Mutation based on Differential Evolution was employed. Furthermore, we redesigned the update method for parameter *a* to better balance exploration and exploitation. The proposed RWOA was evaluated using 23 classical benchmark functions and the impact of six improvement strategies was analyzed. We also conducted a quantitative analysis of RWOA and compared its performance with other state-of-the-art (SOTA) metaheuristic algorithms. Finally, RWOA was applied to nine engineering design optimization problems to validate its ability to solve real-world optimization challenges. The experimental results demonstrated that RWOA outperformed other algorithms and effectively addressed the shortcomings of the canonical WOA.

## 1 Introduction

In recent decades, meta-heuristic algorithms have been developed and widely studied and applied. Due to the complexity and diversity of many real-world problems, traditional exact algorithms often struggle to find the optimal solution in a reasonable amount of time. Meta-heuristic algorithms, on the other hand, find approximate optimal solutions with little computational resources by drawing on problem-specific structure and domain knowledge. They perform well in dealing with complex problems such as large-scale, nonlinear, and multi-modal problems, especially when complete information or accurate explanations are not available at the time of the problem. The following heuristic algorithms have gained great popularity in recent years: the Particle Swarm Algorithm (PSO) [1], the Sparrow Search Algorithm (SSA) [2], the Whale Optimization Algorithm (WOA) [3], the Harris Hawk Optimization Algorithm (HHO) [4], Genetic Algorithms (GA)[5], Simulated Annealing Algorithms (SA) [6], Dung Beetle Optimization Algorithms (DBO) [7], the Grey Wolf Optimizer (GWO) [8], Ant Colony Optimization Algorithm (ACO) [9], Artificial Bee Colony Algorithm (ABC) [10], and so on. Nowadays, meta-heuristic algorithms are widely used to solve life problems such as path planning [11], neural network parameter optimization [12], feature selection [13], optimal scheduling of power systems [14], node coverage optimization of WSN networks [15], shop floor scheduling [16], advanced planning and scheduling (APS) [17], tension/compression spring design [18], welded beam design [19], hydraulic thrust bearing design [20], and antenna design [21] to name a few.

Early engineering design optimization methods mainly include Linear Programming (LP), Nonlinear Programming (NLP), and Integer Programming (IP), focusing on the optimization of a single discipline and usually aiming to solve specific objectives or problems. In these traditional methods, designers mainly rely on experience and intuition to make decisions, using relatively simple strategies to address the problem. As a result, they often achieve a locally optimal solution rather than a globally optimal one. Moreover, traditional design methods typically rely on manual calculations and relatively simple tools, requiring designers to manually adjust parameters. The problem-solving process is cumbersome and time-consuming, and it can only handle simpler models and smaller-scale design spaces. In contrast, modern engineering design optimization emphasizes the use of heuristic algorithms and focuses on the integration of multiple disciplines and global optimization. Modern design methods not only consider a single objective but also take into account the interrelationships between various disciplines and objectives. Through collaborative optimization, Multi-Objective Optimization (MOO), and Multidisciplinary Design Optimization (MDO), designers can achieve more comprehensive and integrated optimization results. Designers can explore a broader design space and seek globally optimal solutions. This paper focuses on the optimization of chemical engineering design using meta-heuristic algorithms, specifically addressing Corrugated bulkhead design, Industrial refrigeration systems, Reactor network design, and piston lever optimization, ordering to enhance chemical plants, productivity, safety, and product quality.

However, meta-heuristic algorithms have some limitations. Particle Swarm Optimization (PSO) faces the risk of premature convergence when handling complex or multi-modal optimization problems, often leading to an early convergence to local optima [1]. Genetic Algorithm (GA), while versatile, exhibits weak local exploitation capabilities and requires tuning of numerous parameters, increasing the complexity of parameter adjustment [5]. The Grey Wolf Optimizer (GWO) is simple in structure and easy to implement, yet in complex or multi-modal problems, GWO may also fall into local optima during later iterations [8]. Harris Hawks Optimization (HHO) is known for its strong exploration and exploitation capabilities, but the complexity of parameter settings makes it challenging to tune, and its effectiveness in addressing complex problems is not guaranteed [4]. Ant Colony Optimization (ACO) performs well in solving discrete and compositional optimization problems, yet the pheromone update mechanism in ACO can lead to premature convergence to local optima [9]. Additionally, ACO requires careful tuning of multiple parameters, such as the pheromone evaporation rate and heuristic factors, and the interactions between these parameters are often complex. The No Free Lunch theorem suggests that the superiority of an optimization algorithm on a specific set of problems does not guarantee its effectiveness across other problem domains. Improving the balance between exploration and exploitation, enhancing the efficiency of the exploration phase, increasing the accuracy of the exploitation phase, and maintaining population diversity in the later stages of iteration have become the major challenges in enhancing the performance of metaheuristic algorithms.

In recent years, many scholars have made various attempts to improve metaheuristic algorithms. [22] In 2021, Ugur Guvenc et al. proposed AGDE. AGDE introduced a mutation operator, an adaptive crossover rate *CR*, and a Fitness-Distance Balance strategy, simulating a more efficient selection mechanism in nature. Its aim was to enhance the balance search capability and diversity of Differential Evolution (DE) [23]. In 2022, Ma C et al. proposed Grey Wolf Optimizer based on the Aquila Exploration Method (AGWO). The AGWO drawn inspiration from Aquila Optimizer (AO), enabling some wolves to possess flying capabilities, thereby expanding the search range and improving global search performance. This modification effectively reduced the risk of getting trapped in local optima. In 2023, Elsisi M et al. proposed the Improved Bald Eagle Search algorithm with dimension learning-based hunting (I-BES), designed to overcome the slow convergence, local optima trapping, and loss of diversity in the early stages that were common issues in the Bald Eagle Search (BES) algorithm [25]. I-BES effectively overcame the tuning issues in model predictive control (MPC) for autonomous vehicles (AVs), including vision dynamics. In 2024, Yang Z et al. proposed Competing leaders Grey Wolf Optimizer (CGWO) [26]. CGWO benefited from a novel mechanism of competing leaders that provided a flexible wolf pack leadership hierarchy to avoid stagnation in local optima and accelerate convergence speed. In addition, a population diversity-enhanced initiation method was designed to help improve the efficiency of the mechanism of competing leaders. These improved metaheuristic algorithms integrated various novel improvement strategies, offering new insights for enhancing the performance of metaheuristic algorithms.

Mirjalili et al. proposed the Whale optimization Algorithm (WOA) in 2016 [3]. WOA is a meta-heuristic algorithm that mimics the feeding strategy of whales, which has the characteristics of strong global optimization ability and simple structure. However, WOA also has certain drawbacks, such as: easy to fall into local optimum, low convergence accuracy, and difficult to balance between global and local exploration. In recent years, scholars have made various attempts to improve WOA. In 2020, Rahnema N et al. proposed the ABCWOA [27]. ABCWOA introduces Random Memory (RM) and Elite Memory (EM) to enhance both convergence and exploration capabilities. In 2020, Ruiye Jiang et al. proposed an improved Whale Optimization Algorithm (WAROA), specifically designed to address complex, large-scale, and constrained optimization problems [28]. The core innovation of WAROA lies in two main adjustments that improve the efficiency and applicability of WOA: first, strategic adjustments of key parameters and the establishment of the basic principles of the original optimization algorithm; second, the introduction of an armed force scheme, which classifies the searching whales and promotes efficient cooperation between different categories. In 2023, Shen Y et al. proposed an improved Whale Optimization Algorithm based on multi-population evolution (MEWOA) [29]. MEWOA divides the population into three subpopulations based on individuals’ fitness: exploration subpopulation, exploitation subpopulation, and moderation subpopulation. Different search strategies are assigned to each subpopulation. This multi-population co-evolution strategy effectively enhances the search capability of WOA. In 2024, Gharehchopogh F S et al. proposed a new hybrid Whale Optimization Algorithm and Golden Jackal Optimization (WOAGJO) aimed at addressing the issue of WOA getting trapped in local optima [30].

## 2 Organization of the paper

Chapter 3 briefly Ooverviewed the major contribution of this research. Chapter 4 primarily analysed the current research works on engineering design. Chapter 5 provided a detailed explanation of the principles of the WOA, along with its advantages and disadvantages. Chapter 6 introduced the RWOA proposed in this paper. Chapter 7 evaluated the performance of RWOA through a series of experiments. Chapter 8 involved testing various metaheuristic algorithms and RWOA on different engineering design optimization problems to validate the practicality and robustness of RWOA.

## 3 Major contributions

The structure of WOA is relatively simple, making it easy to understand and implement. However, WOA struggles to balance exploration and exploitation, and the population quality tends to deteriorate significantly over iterations, leading to insufficient global exploration and premature convergence to local optima. Although the aforementioned studies mentioned in Chapter 1 have improved the performance of WOA to some extent, most of them fail to simultaneously balance exploration and exploitation, enhance convergence speed and accuracy, effectively escape local optima, and maintain a high level of population diversity in the later stages of iteration. Considering WOA performs poor in engineering optimization design, we introduced an enhanced whale algorithm with multi-strategy (RWOA). RWOA aimed to make up for the shortcomings of WOA to a certain extent and explore the potential of WOA as an excellent optimizer for engineering design optimization.

RWOA introduced Good Nodes Set Initialization to generate uniformly distributed populations, employs a newly designed Hybrid Collaborative Exploration (HCE) strategy to enhance global exploration, incorporated Spiral Encircling Prey Strategy that integrates Spiral flight, utilized an Enhanced Spiral Updating Strategy with Levy flight, introduced an Enhanced Cauchy Mutation based on Differential Evolution and introduced a new update mechanism for the parameter *a* to better balance exploration and exploitation. Experiments showed that RWOA effectively addresses the drawbacks of WOA. Furthermore, compared to the classical WOA and other state-of-the-art (SOTA) metaheuristic algorithms, RWOA demonstrated significant advantages in both numerical optimization and real-world optimization problems.

## 4 Research works on engineering design

Based on the complexity and characteristics of optimization problems, optimization methods can generally be divided into two main categories: traditional methods and modern methods. Traditional optimization methods include Linear Programming (LP) and Nonlinear Programming (NLP) [31], which are suitable for problems with small scales and relatively simple objective functions and constraints. Dynamic programming is primarily used for problems involving sequential decision processes, particularly those with time series or multi-stage decisions. Integer Programming (IP) [32] is used for discrete optimization problems, especially when design variables are integers or belong to a finite set.

With the increasing complexity of engineering design, traditional single-discipline optimization methods can no longer meet the demands of modern engineering, thus leading to the emergence of Multi-Disciplinary Design Optimization (MDO). Herskovits et al. proposed numerical models for Simultaneous Analysis and Design Optimization (SAND) and Multi-Disciplinary Design Optimization (MDO), solved using numerical techniques based on the Feasible Arc Interior Point Algorithm (FAIPA) [33]. Even for large-scale optimization problems, this approach significantly reduces computational effort and integrates well with existing engineering simulation codes. The MDO method aims to optimize multiple interdependent disciplines or subsystems simultaneously to achieve more comprehensive and efficient design solutions. In many engineering design problems, such as minimizing weight and maximizing strength. Multiple conflicting objective functions must often be considered. To address these types of problems, Multi-Objective Optimization (MOO) methods have been developed. Yi et al. proposed a method that integrates multi-domain performance criteria into MOO, aiming to provide designers with an integrated optimization solution to improve the overall performance of buildings [34]. MOO methods can simultaneously optimize multiple objectives and provide a set of Pareto optimal solutions.

In recent years, the rapid development of Artificial Intelligence (AI) and Machine Learning (ML) technologies has greatly promote the application of these advanced technologies in engineering design optimization. For example, Pablo N. Pizarro et al. used deep neural networks to predict wall thickness and length based on previous architectural and engineering projects, improving design efficiency and reducing trial-and-error processes [35]. Fang et al. proposed a deep reinforcement learning (DRL)-based wind turbine rotor speed optimization method, considering rain intensity and wind speed conditions to reduce rain erosion-induced blade coating fatigue damage [36]. Furthermore, data-driven optimization, using big data technology, extracts valuable information from historical data, learns optimization patterns, and provides scientific data support for the design process. The application of AI and ML technologies not only enhances the efficiency and accuracy of the optimization process but also provides new momentum and perspectives for engineering design innovation.

Compared to traditional methods, modern optimization methods rely more on heuristic algorithms to tackle complex, multi-modal, and highly constrained problems. Typical modern optimization methods include Genetic Algorithms (GA), which are based on principles of biological evolution and are especially effective for complex and nonlinear problems [5]; Particle Swarm Optimization (PSO), inspired by the foraging behavior of bird flocks, with strong global search capabilities [1]; Simulated Annealing (SA), which mimics the physical annealing process to avoid local optima, widely used in complex system optimization [6]; and Ant Colony Optimization (ACO), which simulates the foraging behavior of ants and is particularly useful for path optimization problems [9]. Additionally, topology optimization is widely applied in structural design to optimize the shape and material distribution of structures, thereby improving design efficiency and performance.

## 5 WOA

The WOA is a new meta-heuristic algorithm proposed by Mirjalili et al. from Griffith University, Australia, which mimics the behaviour of whales in searching for, encircling, and capturing their prey for the purpose of solving a complex optimization problem [3].

### 5.1 Initialization

As shown in Fig 2 on the left, like most meta-heuristic algorithms, WOA uses pseodo-random number initialization for population initialization.


$$\begin{array}{ccc} X_{i,j}=(ub-lb)\cdot Rand+lb & & \end{array}$$ (1)

where $X_{i,j}$ is whale population initialized by pseodo-random number initialization; *ub* and *lb* are the upper limit and lower limit of the problem; *Rand* is a random number between 0 and 1.

### 5.2 Encircling prey

Humpback whales recognize the location of their prey and encircle them. Since the location of the optimal design in the search space is unknown, WOA assumes that the current optimal candidate solution is the target prey or a near-optimal solution. After defining the optimal search agent, other search agents will try to update their positions to the optimal search agent. This behavior is represented by Eqs 2 and 3.


$$\begin{array}{c} D=|C\cdot X^{*}(t)-X(t)| \end{array}$$ (2)

$$\begin{array}{c} X(t+1)=X^{*}(t)-A\cdot D \end{array}$$ (3)

where *t* is the current iteration; *A* and *C* are coefficient vectors; $X^{*}$ is the position of the current best solution; *X* is the position of the whale.

If the result of each iteration has a better solution, the fitness value of this position is smaller than the fitness value of this position, then the position vector at this time will be set as the new $X^{*}$.

**Fig 1:**
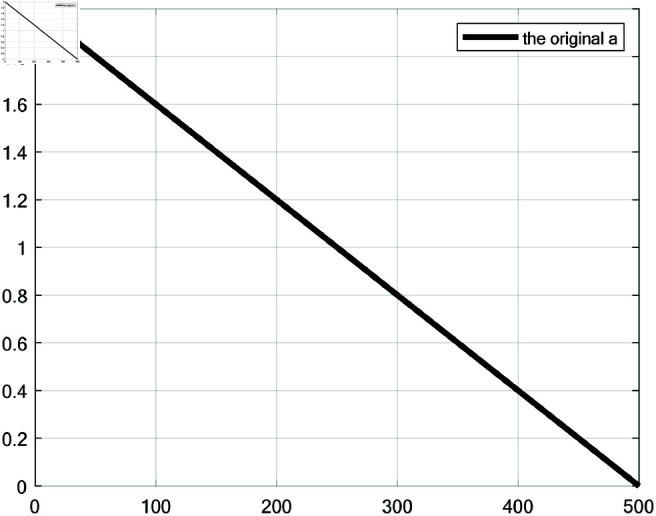
The update method of parameter *a* in the original WOA.

The formulae for vector *A* and *C* are given below:


$$\begin{array}{c} A=2a\cdot r-a \end{array}$$ (4)

$$\begin{array}{c} a=2-2\cdot \frac{t}{T} \end{array}$$ (5)

$$\begin{array}{c} C=2\cdot r \end{array}$$ (6)

where *r* is a vector of random numbers from 0 to 1; *a* decreases from 2 to 0 during the iteration, as shown in Fig 1; *t* is the current number of iterations; *T* is the maximum number of iterations.

### 5.3 Bubble-net attacking method

In addition to encircling their prey, whales also use the bubble net attack method to attack their prey. They trap their prey by blowing round bubbles in the water to create a water net. At around 15 meters underwater, the whales swim upwards in a spiral position and spit out bubbles of varying sizes to encircle their prey and bring them closer to a central area. The whale then swallows the prey in the bubble net in a near vertical position. Therefore, the bubble net attack method is divided into the following two strategies:

1. **Shrinking encircling**: This behaviour is achieved by reducing the value of *A* in Eq 4. The position is updated as shown in Eq 3.
2. **Spiral updating position**: The method first calculates the whale position *X* and the prey position $X^{*}$ in a straight line distance $D^{\prime }$ from the prey position as shown in Eq 7. And then creates a spiral equation to simulate the whale spiraling up to encircle the prey as shown in Eq 8.
  $$\begin{array}{c} D^{\prime }=|X^{*}(t)-X(t)| \end{array}$$ (7)
  $$\begin{array}{c} X(t+1)=D^{\prime }\cdot e^{bl}\cdot \cos(2\pi l)+X^{*}(t) \end{array}$$ (8)
  $$\begin{array}{c} a_{1}=-1-\frac{t}{T} \end{array}$$ (9)
  $$\begin{array}{c} l=(a_{1}-1)\cdot Rand+1 \end{array}$$ (10)
  where *b* is a constant defining the shape of the logarithmic helix, usually set to 1; $a_{1}$ is a linearly varying parameter of [-2, -1]; *t* is the current number of iterations; *T* is the maximum number of iterations; *Rand* is a random number between 0 and 1; the spiral coefficient *l* takes the values [-2, 1].

WOA sets a 50% probability for each of the Shrinking encircling mechanism and spiral updating position mechanism of the whale updating its position as follows:


$$\begin{array}{ccc} X(t+1)=\{\begin{array}{cc} X^{*}(t)-A\cdot D\; & p<0.5\\ D^{\prime }\cdot e^{bl}\cdot cos(2\pi l)+X^{*}(t)\; & p\geq 0.5 \end{array} & & \end{array}$$ (11)

### 5.4 Search for prey

If the whale moves beyond the position where the prey exists, then the whale will abandon the previous moving direction and randomly search for other prey in other directions to avoid falling into a local optimum. The modeling of the whale searching for prey is as follows:


$$\begin{array}{ccc} D^{\prime \prime }=|C\cdot X_{rand}-X(t)| & & \end{array}$$ (12)

$$\begin{array}{ccc} X(t+1)=X_{rand}-A\cdot D^{\prime \prime } & & \end{array}$$ (13)

where $X_{rand}$ is a random whale chosen from the current population; *A* and *C* are described in Eq 4 and Eq 6.

### 5.5 Advantages and disadvantages of WOA

The pseudo-code of the WOA is shown in Algorithm 1.

**Algorithm 1**: WOA

**Begin**

(*Pseodo-random number initialization*)

Initialize the population using Pseodo-random number method;

Initialize the parameters  ( *T* , *N* , *p* , etc. ) ;

Calculate the fitness of each search agent;

The best search agent is $X^{*}$;

**while** *t* < *T*

**for** each search agent

Update *a*, *A*, *C*, *l*, and *p*;

**if** *p* < 0 . 5

**if**  | *A* | < 1

(*Search for prey*)

Update the position of the current search agent by      Eq 3;

**else**

(*Encircling prey*)

Update the position of the current search agent by      Eq 13;

**end if**

**else**

(*Spiral updating*)

Update the position of the current search agent by      Eq 8;

**end if**

**end for**

Check if any search agent goes beyond the search space and amend it;

Calculate the fitness of each search agent;

Update $X^{*}$ if there is a better solution;

*t* = *t* + 1

**end while**

**return** $X^{*}$

**End**

From Algorithm 1, it can be seen that the late iteration of traditional WOA causes the WOA to have the disadvantages of weak global search ability, slow convergence speed, low accuracy of searching for the best and easy to fall into the local optimum due to the reduction of the diversity of the population. The development and exploration ability of traditional WOA is weak, and the parameters of WOA are difficult to balance the global exploration and local development of the whale algorithm. In addition, the traditional WOA does not take into account the possible differences in the guiding force of the prey to guide the whale for position updating. WOA has much room for improvement. Therefore, we proposed RWOA to address the above shortcomings of WOA.

## 6 RWOA

### 6.1 Good nodes set

**Fig 2:**
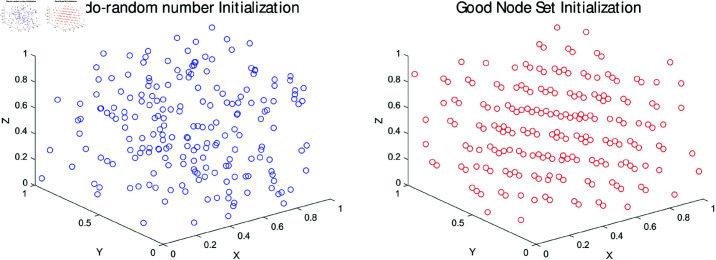
Pseodo-random number initialization vs good nodes set initialization.

The traditional WOA uses Pseodo-random number initialization to generate the population, which is simple, direct and random, but the randomly generated population is not uniformly distributed in the whole solution space, it is denser somewhere and sparser somewhere, which can’t achieve the uniform distribution of the population in the whole search space, and it leads to the inefficiency of WOA in searching process due to the low quality of the population as shown in Fig 2 on the left. Therefore, RWOA uses Good Nodes Set Initialization to generate uniformly distributed populations.

The concept of the Good Nodes Set was first proposed by the Chinese mathematician Loo-keng Hua. This method constructs a set of nodes that are uniformly distributed in the space, which provides a significant advantage over random initialization, especially in higher-dimensional spaces. The key intuition behind this approach is that the construction of the Good Nodes Set is independent of the number of dimensions, which ensures uniformity in any dimensional space. In contrast, random initialization often suffers from clustering of nodes in some regions and large gaps in others, especially as the dimensionality increases. The uniform distribution provided by the Good Nodes Set improves the search efficiency by avoiding such issues and facilitating better global exploration.

Mathematically, the Good Nodes Set is defined as a sequence of nodes in a unit hypercube *D*-dimensional Euclidean space, where the nodes are spaced according to a specific non-random, deterministic pattern. The uniformity of the distribution can be understood through the fractional part of the product ${kr}^{d}$, ensuring that the nodes are spread evenly. This deterministic nature contrasts with the random nature of pseudo-random number initialization, where the nodes are arbitrarily distributed, leading to inefficiencies.

In this sense, Good Nodes Set Initialization enhances the population quality by providing a more structured and evenly distributed set of candidate solutions, improving both the exploration and exploitation abilities of the algorithm, as illustrated in Fig 2 on the right [37]. Good Nodes Set in *D*-dimensional space is described by Eq 14 as follows:


$$\begin{array}{ccc} P_{r}^{M}=\{p(k)=(\{kr\},\{kr^{2}\},\ldots ,\{kr^{D}\})|k=1,2,\ldots ,M\} & & \end{array}$$ (14)

where {*x*} represents the fractional part of *x*; *M* is the number of points; *r* is a deviation parameter greater than zero; the constant *C* ( *r* , *𝜀* )  is associated only with *r* and *𝜀* is related to and is a constant greater than zero.

This set $P_{r}^{M}$ is called Good Nodes Set and each node *p*(*k*) in it is called a Good Node. Assume that the upper and lower bounds of the $i^{th}$ dimension of the search space $X_{max}^{i}$ are and $X_{min}^{i}$, then the mapping formula for mapping the Good Nodes Set to the actual search space is:


$$\begin{array}{ccc} X_{k}^{i}=x_{min}^{i}+p_{i}(k)\cdot (X_{max}^{i}-X_{min}^{i}) & & \end{array}$$ (15)

### 6.2 Hybrid collaborative exploration (HCE) strategy

Red-billed Blue Magpie Optimization algorithm (RBMO) is a new meta-heuristic algorithm proposed by Shengwei Fu et al. in 2024 [38], which has a powerful search capability by simulating the hunting and food storage behaviour of red-billed blue magpies. Red-billed blue magpies use two strategies to search for food, and they usually act in small groups of 2-5 individuals or in groups of more than 10 individuals to improve the search efficiency.

Location updates were made using Eq 16 when red-billed blue magpies were searching for food in small groups:


$$\begin{array}{ccc} X(t+1)=X(t)+(\frac{1}{p}\cdot \overset{p}{\underset{m=1}{\sum }}X^{m}(t)-X^{rs}(t))\cdot Rand_{1} & & \end{array}$$ (16)

where *t* represents the current number of iterations; *X* represents the location of the search agent; *p* represents the number of red-billed blue magpies in 2-5 cliques randomly selected from all search individuals; $X^{m}$ represents the $m^{th}$ randomly selected individual; $X^{rs}$ represents the randomly selected search agent in the current iteration.

Location updates were made using Eq 17 when red-billed blue magpies were searching for food as a group:


$$\begin{array}{ccc} X(t+1)=X(t)+(\frac{1}{q}\cdot \overset{q}{\underset{m=1}{\sum }}X^{m}(t)-X^{rs}(t))\cdot Rand_{2} & & \end{array}$$ (17)

where *q* represents the number of red-billed blue magpies in 10-*n* randomly selected cliques from all searched individuals and *n* is the population size.

**Fig 3:**
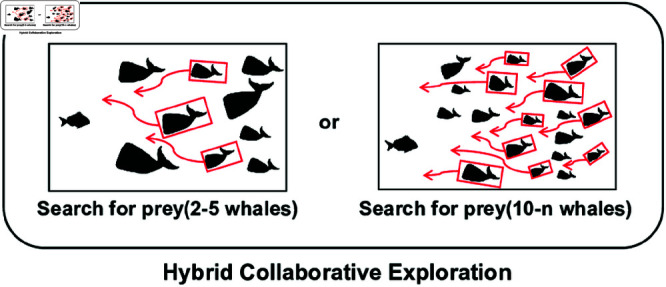
Hybrid collaborative exploration mechanism. In the search for prey, whales adopt the cooperative strategy of the red-billed blue magpies, randomly selecting 2-5 whales or 10-*n* whales to search for prey.

In the search-for-prey phase, the WOA uses only the position of a randomly selected whale for searching, which may limit the diversity of search paths, especially in high-dimensional search spaces, and may lead to insufficient search capability. In addition, although WOA updates the position by randomly selecting a reference individual, this single randomness may not be sufficient to help the WOA explore regions far away from the current optimal solution, which reduces the global search capability. Therefore, Hybrid Collaborative Exploration mechanism in RWOA incorporates the idea of searching for food from RBMO algorithm by randomly selecting a small group of 2-5 whales or a group of 10-*n* whales in the whale population to search for food randomly, as shown in Fig 3. By introducing the multi-option positions of 2-5 or 10-*n* whales for reference, RWOA is able to combine the information of multiple individuals for position updating. This means that more directions and paths are considered in the RWOA position update, avoiding the limitations of a single position update and allowing it to jump and explore over a larger area while searching. This strategy of incorporating RBMO helps to enhance the global search capability of WOA, enabling the algorithm to better discover the global optimal solution. In addition, this strategy takes into account the position of the current best solution, effectively guiding the search of the whales towards better solutions. As the number of iterations increases, the reliance on the current best solution’s position gradually decreases. This means that, in the early stages, the position update is more influenced by the global best solution to guide the search, while as iterations progress, the reliance on the current best solution’s position diminishes, the search range expands, and greater emphasis is placed on diversity, thereby preventing premature convergence to local optima. This multi-option reference reduces the instability caused by a single random individual, improves the robustness of the algorithm, and makes WOA perform more stably in the face of various optimization problems.

The formula for Hybrid Collaborative Exploration (HCE) strategy of RWOA is shown in Eq 18:


$$\begin{array}{ccc} X(t+1)=\{\begin{array}{c} (1-\frac{t}{T})\cdot X^{*}(t)+A\cdot \left(\frac{1}{p}\cdot \overset{p}{\underset{m=1}{\sum }}X^{m}(t)-X^{*}(t)\right)\cdot Rand_{1},\;r<0.5\;\\ (1-\frac{t}{T})\cdot X^{*}(t)+A\cdot \left(\frac{1}{q}\cdot \overset{q}{\underset{m=1}{\sum }}X^{m}(t)-X^{*}(t)\right)\cdot Rand_{2},\;r\geq 0.5\; \end{array} & & \end{array}$$ (18)

where *A* is the vector of coefficients; $X^{*}$ is the position of the current best solution; *p* and *q* represent the number of whales in 2-5 small groups or 10-*n* flocks randomly selected from all searched individuals respectively; $Rand_{i}$ denotes a random number from 0 to 1.

### 6.3 Spiral encircling prey strategy

As shown in Eq 3, the original WOA uses an encircling prey strategy, where the whale’s position is updated based on the distance to the prey. While effective in approaching the optimal solution, this strategy can lead to premature convergence and local optima, particularly in complex, multi-modal problems. The simplicity of the strategy limits exploration during local search and makes the algorithm sensitive to initial positions.

**Fig 4:**
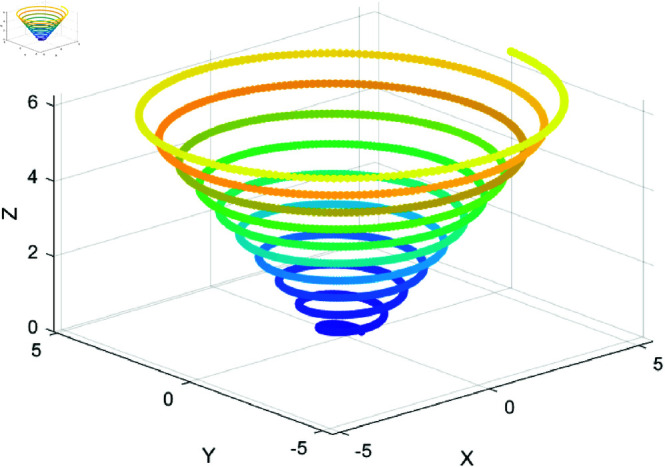
Simulation of spiral flight.

**Fig 5:**
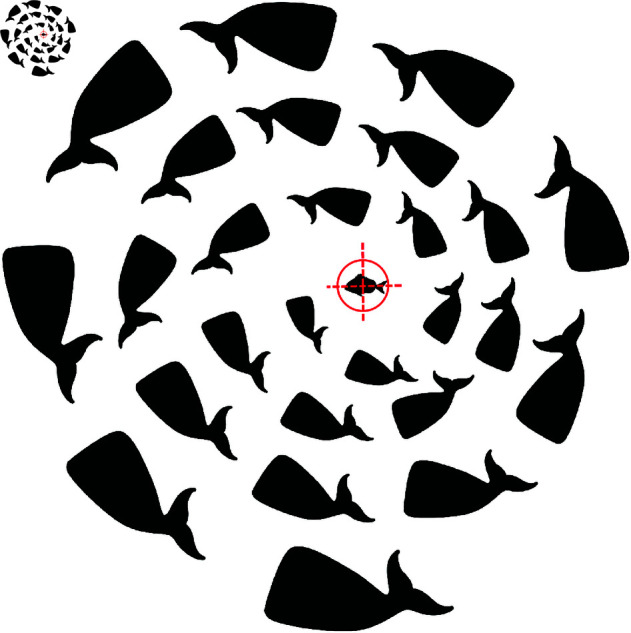
Spiral encircling prey mechanism. The whales encircle the prey in a spiral pattern.


Spiral flight is a continuous and systematic search pattern that differs from traditional linear or local search methods, as shown in Fig 4. It allows the whales to generate more varied trajectories while encircling the prey. This spiral motion not only enhances the encircling capability towards the target but also helps avoid local convergence during the search process. The introduction of spiral motion improves the whales’ precision while encircling the prey, particularly when approaching the optimal solution. The nonlinear spiral trajectory makes the search trajectory more flexible, ensuring that the whale maintains ongoing exploration of the target while preventing quick convergence to a particular location, which may cause it to miss other potential optimal solutions. By incorporating the spiral strategy, the whales’ movement direction and distance become more diverse, enabling exploration of the solution space around the prey in multiple directions, thus enhancing the diversity of the search process. Fig 5 is a schematic diagram of the Spiral Encircling Prey mechanism.

$$\begin{array}{c} X(t+1)=X^{*}(t)+e^{Z\cdot L}\cdot cos(2\pi L)\cdot |A\cdot D| \end{array}$$ (19)

$$\begin{array}{c} L=2\cdot r-1 \end{array}$$ (20)

$$\begin{array}{c} Z=e^{cos(\pi \cdot (1-\frac{t}{T}))} \end{array}$$ (21)

where *r* is a random number from 0 to 1; *Z* and *L* are spiral coefficients.

### 6.4 Enhanced spiral updating strategy

While the original spiral updating strategy of WOA aids in local search, the lack of randomness and perturbation mechanisms may cause whale individuals to converge around the leader (current best solution) in a short time, limiting the ability to escape local optima. To address this, this paper proposed an Enhanced Spiral Updating strategy by introducing Levy flight into the Spiral Updating strategy.

**Fig 6:**
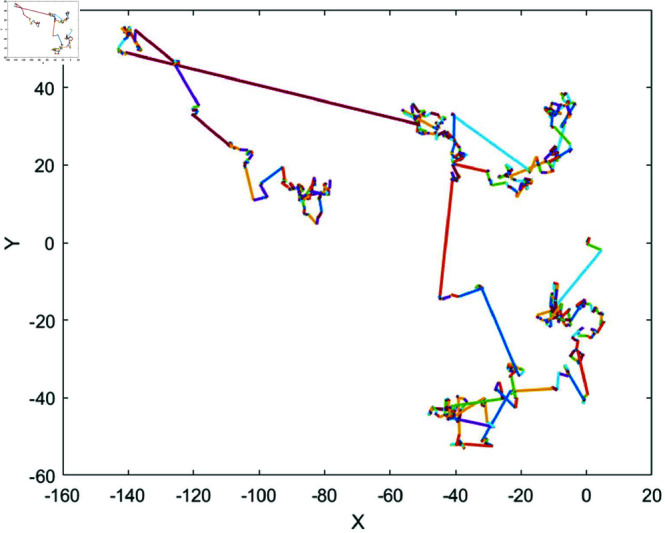
Simulation of Levy flight.

Levy flight is a random process whose distribution characteristics lie between Brownian motion and more extreme jumping behaviors. The concept originated from the work of the mathematician Paul Levy in the 1920s. Inspired by foraging behavior in nature and jumping phenomena in complex systems, Levy flight combines short-range exploration with long-distance jumps, resembling the foraging paths of predators such as sharks, birds, and insects. Fig 6 illustrates a simulation of Levy flight. It is a random walk model based on the Levy distribution, which has a higher probability of producing longer step sizes, enabling whales to explore vast distances through jumps. In global search scenarios, Levy flight allows for large jumps, which helps avoid getting trapped in local optima, while still maintaining some local search capability through shorter steps. By incorporating unequal probabilities for both long and short jumps, Levy flight enhances the diversity of the search process, making it better suited to adapt to more complex solution space structures. The step size *L*(*s*) of Levy flight is calculated as follows:


$$\begin{array}{ccc} L(s)=\frac{u}{|\nu {|}^{\frac{1}{\beta }}} & & \end{array}$$ (22)

where *u* and *ν* are normally distributed; *β*=1.5.


$$\begin{array}{c} u∼N(0,\sigma_{u}^{2}) \end{array}$$ (23)

$$\begin{array}{c} v∼N(0,1) \end{array}$$ (24)

The calculation of $\sigma_{u}$ is given by:


$$\begin{array}{ccc} \sigma_{u}={\left(\frac{\Gamma (1+\beta )\cdot \sin\left(\frac{\pi \beta }{2}\right)}{\Gamma \left(\frac{1+\beta }{2}\right)\cdot \beta \cdot 2^{\frac{\beta -1}{2}}}\right)}^{\frac{1}{\beta }} & & \end{array}$$ (25)

By introducing Levy flight, the long step sizes inherent in the Levy flight mechanism help individuals leap freely across the entire search space. This significantly boosts the jumping capability of the Spiral Updating strategy, thereby increasing the chance of escaping local optima and enhancing the global search ability. The introduction of Levy flight allows whales to not only follow the spiral trajectory for local search but also utilize larger jumps for global exploration, which helps prevent the algorithm from converging prematurely to local optima. This strategy enhances the diversity and flexibility of whale movements, particularly in cases where the search space is large and the solution space is complex, effectively avoiding premature convergence. Enhanced Spiral Updating strategy was modeled as follows:


$$\begin{array}{ccc} X(t+1)=L(s)\cdot X^{*}(t)+D^{\prime }\cdot e^{bl}\cdot \cos(2\pi l) & & \end{array}$$ (26)

where $X^{*}(t)$ represents the position of the current best solution; *L*(*s*) is the step size of the Levy flight; *b* is a constant that defines the shape of a logarithmic spiral, usually set to 1; the value of spiral coefficient *l* is  [ − 2 , 1 ] , and *l* is calculated in Eq 10; *X* is the whale’s position; $a_{1}$ is the parameter of the linear change of  [ − 2 , − 1 ] , calculated in Eq 9; *Rand* is a random number between 0 and 1.

### 6.5 Enhanced cauchy mutation based on differential evolution

The standard position update strategies for WOA mainly rely on bionic principles, such as prey encirclement and spiral update. These strategies are usually more focused on searching in the vicinity of the current optimal solution, especially in the later stages of the iteration, the WOA may gradually converge to a particular region, leading to a decrease in the exploration ability of the search space. By introducing a mutation strategy after the position update, a new perturbation can be introduced into the solution space, generating a certain degree of mutation that allows individual whales to jump out of the current local optimal region and explore a wider solution space. In addition, for algorithms like WOA that rely on optimal solutions in the population for search, the variation strategy can help individuals jump out of the locked region so that the solution of WOA does not fall into a local optimum, especially when dealing with complex problems with multiple local optimal solutions. By incorporating a variation strategy into the WOA, the possible limitations of the WOA in the later convergence phase can be compensated for, ensuring that the WOA continues to explore rather than converging to a sub-optimal solution early on. We incorporate a novel Cauchy mutation strategy based on Differential Evolution into RWOA.

First, we generate an intermediate solution $X_{new}^{\prime \prime }$ by Differential Evolution.


$$\begin{array}{ccc} X_{new}^{\prime \prime }(t+1)=X_{i}(t)+F\cdot ((X^{*}(t)-X_{D}(t))+(X_{E}(t)-X_{F}(t))) & & \end{array}$$ (27)

where $X_{i}$ represents the current position of the $i^{th}$ individual; $X^{*}$ represents the position of the current best individual; $X_{D}$, $X_{E}$ and $X_{F}$ represent the positions of three different individuals randomly selected from the population respectively; *F* is the factor controlling the scaling of the difference vector, which is calculated as in Eq 27.


$$\begin{array}{ccc} F=1+tan(\pi \cdot (Rand-0.5)) & & \end{array}$$ (28)

where *Rand* denotes a random number between 0 and 1.

Subsequently, execute Cauchy mutation on the generated intermediate solutions $X_{new}^{\prime }$:


$$\begin{array}{ccc} X_{new}^{\prime }(t+1)=X_{new}^{\prime \prime }(t)\cdot (1+\delta ) & & \end{array}$$ (29)

where *δ* denotes the perturbation term sampled from the Cauchy distribution:


$$\begin{array}{ccc} \delta ∽Cauchy(0,1) & & \end{array}$$ (30)

Then, after applying the mutation operation to the intermediate solution, boundary checking and adjustment are required, otherwise the population degradation of WOA may occur. The boundary checking is performed as follows:


$$\begin{array}{ccc} X_{new}(t+1)=min(max(X_{new}^{\prime }(t),lb),ub) & & \end{array}$$ (31)

where *ub* and *lb* represent the upper and lower bounds of the problem respectively.

Finally, if the mutated solution $X_{new}$ has a better fitness than the original solution $X_{i}$, then the solution is updated with $X_{new}$.

### 6.6 Redesign of parameter *a*

**Fig 7:**
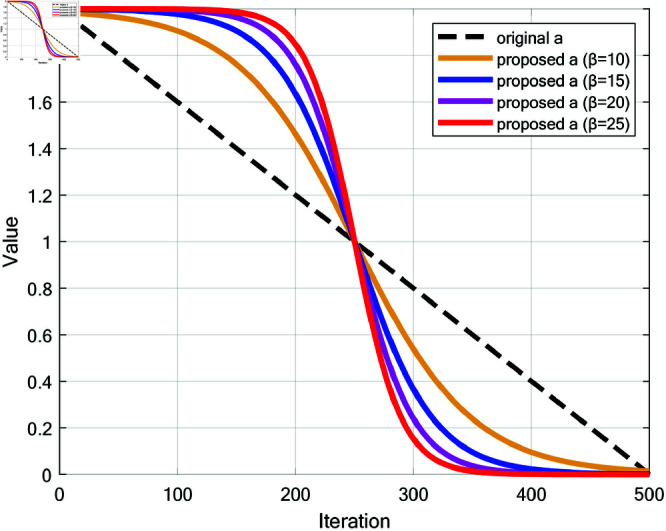
Comparison between the update methods of the original parameter *a* and the proposed *a* (*β*=10, *β*=15, *β*=20 and *β*=25).

Although integrating these strategies improves WOA’s performance, the traditional linear update of parameter *a* from 2 to 0 no longer meets RWOA’s needs. The linear method results in a smooth behavior change, especially at later stages, where the minimal reduction slows convergence. To address this, a Sigmoid-based parameter *a* update was proposed to balance global exploration and local exploitation, as shown in Eq 32. Fig 7 compares the proposed Sigmoid method with the original linear one.

The Sigmoid update reduces *a* slowly at first, rapidly in the middle, and slowly again at later stages. This simulates complex variation, enabling distinct convergence behaviors: slow early reduction supports broad exploration, rapid mid-stage decrease enhances convergence speed, and slower late-stage reduction balances exploration and exploitation for fine local searches. This strategy improves adaptability and final solution accuracy, making the algorithm more effective and reliable for complex problems.


$$\begin{array}{ccc} a=2-\frac{2}{1+e^{-\beta (\frac{t}{T}-0.5)}} & & \end{array}$$ (32)

where *β* is the scaling factor of the Sigmoid function and value of *β* was tested in the parameter sensitivity analysis experiment; *t* is the current iteration number; *T* is the maximum number of iterations.

The pseudo-code of the complete RWOA is provided in Algorithm 2.

**Algorithm 2**: RWOA

**Begin**

(*Good Nodes Set Initialization*)

Initialize the population using Good Nodes Set method;

Initialize the parameters  ( *T* , *N* , *p* , etc. ) ;

Calculate the fitness of each search agent;

The best search agent is $X^{*}$;

**while** *t* < *T*

**for** each search agent

Update *a*, *A*, *C*, *l*, and *p*;

**if** *p* < 0 . 5

**if**  | *A* | < 1

(*Spiral Encircling Prey*)

Update the position of the current search agent by Eq 19;

**else**

(*Hybrid Collaborative Exploration*)

Update the position of the current search agent by Eq 18;

**end if**

**else**

(*Enhanced Spiral Updating*)

Update the position of the current search agent by Eq 26;

**end if**

**end for**

(*Enhanced Cauchy Mutation based on Differential Evolution*)

**for** each search agent

Generate an intermediate solution $X_{new}^{\prime \prime }$ by DE through Eq 27;

Execute Cauchy mutation to generate an another intermediate

solution

$X_{new}^{\prime }$ by Eq 29;

**if** $X_{new}^{\prime }$ is within the boundary

$X_{new}$ = $X_{new}^{\prime }$;

**else**

**if** $X_{new}^{\prime }$ exceeds the upper boundary *ub*

set it to the upper boundary *ub*;

**else if** $X_{new}^{\prime }$ exceeds the lower boundary *lb*

set it to the lower boundary *lb*;

**end if**

**end if**

**end for**

Calculate the fitness of each search agent;

Update $X^{*}$ if there is a better solution;

*t* = *t* + 1

**end while**

**return** $X^{*}$

**End**

### 6.7 Time complexity analysis

Assume that the time complexity of Pseodo-random number initialization in WOA is *O*(*ND*). During each iteration, and the total time complexity of position updates is *O*(*ND*). Therefore, the total time complexity per iteration is *O*(*ND*). If the algorithm iterates *T* times, the total time complexity of WOA is calculated as:

Total Time Complexity 1 = Initialization + *T* * (the total time complexity per iteration) = *O*(*ND*) + *T* * *O*(*ND*) = *O* ( *T* ∗ *ND* ) 

Assume that the time complexity of Good Nodes Set Initialization in RWOA is *O*(*ND*). During each iteration, and the total time complexity of position updates is *O*(*ND*), the time complexity of Enhanced Cauchy Mutation based on Differential Evolution is *O*(*ND*), Therefore, the total time complexity per iteration is *O*(*ND*). If the algorithm iterates *T* times, the total time complexity of RWOA is calculated as:

Total Time Complexity 2 = Initialization + *T* * (the total time complexity per iteration) =*O*(*ND*) + *T* * *O*(*ND*) = *O* ( *T* ∗ *ND* ) 

In summary, the time complexity of RWOA and WOA are the same, both are *O* ( *T* ∗ *ND* ) .

## 7 Simulation experiments and analysis

The experimental environment for experiments was Windows 11 (64bit), Intel(R) Core(TM) i5-8300H CPU @ 2.30GHz, 8GB running memory and the simulation platform is Matlab R2023a.

In order to validate the performance and effectiveness of the RWOA, the following four experiments are designed to test the algorithms on selected classical benchmark functions [39]:

- **Experiment 1:** Each of the six improvement strategies was removed from RWOA respectively and an ablation study was performed on the 23 classical benchmark functions in Table 2;
- **Experiment 2:** An parameter sensitivity analysis experiment was performed and the four values of the scaling factor *β* in Eq 32 were tested on the benchmark functions to determine the optimal value of *β* that best balanced the exploration and exploitation capabilities of the RWOA;
- **Experiment 3:** A qualitative analysis experiment was performed by applying RWOA on the benchmark functions to comprehensively evaluate the performance, robustness and exploration-exploitation balance of RWOA in different types of problems, by assessing search behavior, exploration-exploitation capability and population diversity;
- **Experiment 4:** RWOA was tested against other state-of-the-art metaheuristic algorithms (basic metaheuristic algorithms and enhanced metaheuristic algorithms) on the classical benchmark functions, to verify the superiority of RWOA.

**Table 1:** Current research on improved metaheuristic algorithms.

Algorithm	Year	Author	Source of Inspiration
ABCWOA [27]	2020	E Ugur Guvenc et al.	Random Memory (RM) and
			Elite Memory (EM).
WAROA [28]	2020	Ruiye Jiang et al.	Armed force program
			and strategic adjustment.
AGDE [23]	2021	E Ugur Guvenc et al.	Mutation operator, an adaptive
			crossover rate and FDB strategy.
AGWO [24]	2022	Ma C et al.	Exploration phase of AO.
I-BES [25]	2023	Elsisi M et al.	Dimension learning-based hunting.
MEWOA [29]	2023	Shen Y et al.	Subpopulations based on
			individuals’ fitness.
CGWO [26]	2024	Yang Z et al.	Mechanism of competing leaders
WOAGJO [30]	2024	Gharehchopogh F S et al.	The idea of Golden
			Jackal Optimization (GJO).

**Table 2:** Classical benchmark functions [39].

Function	Function’s Name	Type	Dimension	Best Value
F1	Sphere	Uni-modal	30	0
F2	Schwefel’s Problem 2.22	Uni-modal	30	0
F3	Schwefel’s Problem 1.2	Uni-modal	30	0
F4	Schwefel’s Problem 2.21	Uni-modal	30	0
F5	Generalized Rosenbrock’s Function	Uni-modal	30	0
F6	Step Function	Uni-modal	30	0
F7	Quartic Function	Uni-modal	30	0
F8	Generalized Schwefel’s Function	Multi-modal	30	-12569.5
F9	Generalized Rastrigin’s Function	Multi-modal	30	0
F10	Ackley’s Function	Multi-modal	30	0
F11	Generalized Griewank’s Function	Multi-modal	30	0
F12	Generalized Penalized Function 1	Multi-modal	30	0
F13	Generalized Penalized Function 2	Multi-modal	30	0
F14	Shekel’s Foxholes Function	Multi-modal	2	0.998
F15	Kowalik’s Function	Multi-modal	4	0.0003075
F16	Six-Hump Camel-Back Function	Compositional	2	-1.0316
F17	Branin Function	Compositional	2	0.398
F18	Goldstein-Price Function	Compositional	2	3
F19	Hartman’s Function 1	Compositional	3	-3.8628
F20	Hartman’s Function 2	Compositional	6	-3.32
F21	Shekel’s Function 1	Compositional	4	-10.1532
F22	Shekel’s Function 2	Compositional	4	-10.4029
F23	Shekel’s Function 3	Compositional	4	-10.5364

### 7.1 Ablation study

**Fig 8:**
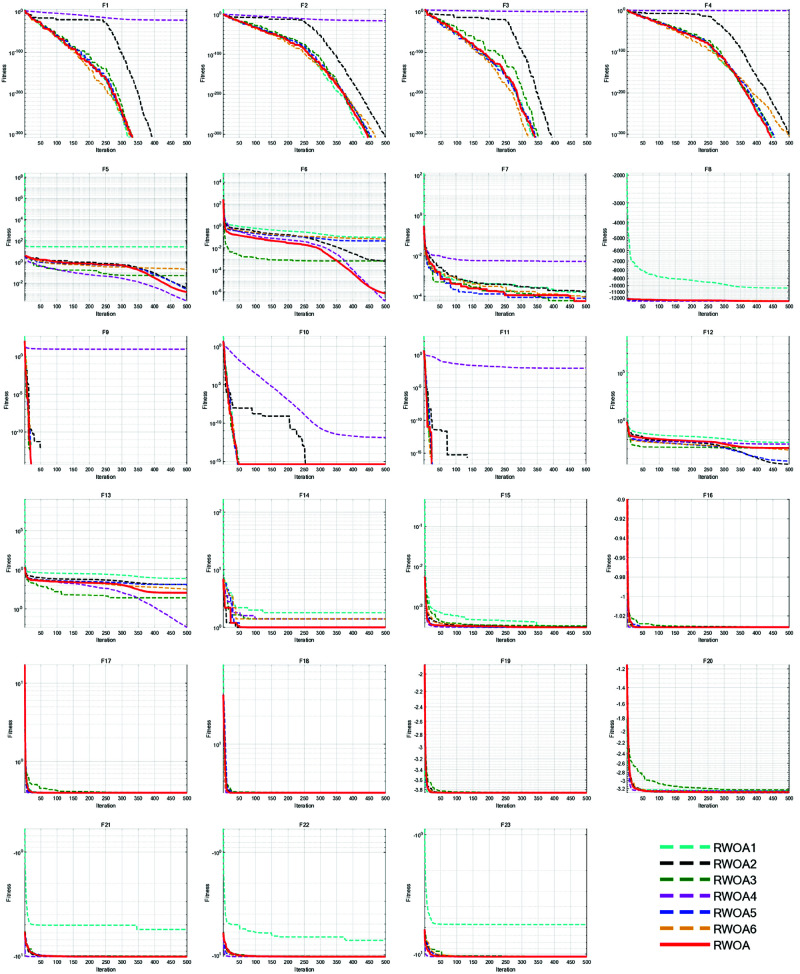
Iteration curves of the algorithms in ablation study.

In this ablation study, we excluded each of the six improvement strategies from the RWOA:

- RWOA1: RWOA without Good Nodes Set Initialization;
- RWOA2: RWOA without Hybrid Collaborative Exploration strategy;
- RWOA3: RWOA without Spiral Encircling Prey strategy;
- RWOA4: RWOA without Enhanced Spiral Updating strategy;
- RWOA5: RWOA without Enhanced Cauchy Mutation based on Differential Evolution;
- RWOA6: RWOA with the original update method of parameter *a*;

The number of iterations *T*=500 and the number of populations *N*=30 were set uniformly. Each algorithm was run 30 times individually on 23 classical Benchmark Functions for performance analysis. The Friedman values of the algorithms were recorded. And the iteration curves were shown in Fig 8.

As shown in Fig 8, the Good Nodes Set Initialization generated whale individuals that were more uniformly distributed in the solution space. This high-quality population provided a significant advantage when addressing problems such as F5-F6, F12-F15, and F21-F23. The Hybrid Collaborative Exploration strategy integrated RBMO’s cluster hunting strategy, allowing RWOA to update positions by incorporating the positional information of multiple individual whales. This avoided the limitations associated with single-position updates, enabling RWOA to explore a larger search range. Consequently, the global search capability of WOA was enhanced, strengthening its ability to solve complex functions. The Spiral Encircling Prey Strategy, incorporating a nonlinear step size through Spiral flight, increased the randomness and introduced nonlinear fluctuations in position updates. This addition brought both periodicity and unpredictability to the algorithm, allowing it to consistently avoid local optima and prevent premature convergence, particularly when tackling complex problems such as F5-F6 and F15-F17. The Enhanced Spiral Updating strategy, which introduced Levy flight, allowed whale individuals to effectively escape local optima while spiraling upward. The incorporation of step size of Levy flight enhanced the algorithm’s global exploration ability and significantly improved its convergence speed. This strategy is particularly advantageous when dealing with functions such as F1-F4 and F9-F11. The Enhanced Cauchy Mutation based on Differential Evolution integrated the concepts of Differential Evolution and Cauchy mutation, helping the algorithm to generate superior solutions after position updates. The new update method for parameter *a* based on the Sigmoid function, effectively balanced exploration and exploitation. This novel convergence behavior improved the algorithm’s adaptability and optimization precision. As shown in Table 3, the average Friedman value of RWOA is 2.9217, ranking first. This indicates that RWOA was the optimal choice.

**Table 3:** Friedman values of the algorithms in the ablation study.

Metrics	RWOA1	RWOA2	RWOA3	RWOA4	RWOA5	RWOA6	RWOA
Average Friedman Value	4.6239	3.5609	5.0652	3.5891	3.5848	4.5022	**2.9217**
Rank	5	2	7	4	3	6	**1**

### 7.2 Parameter sensitivity analysis experiment

The purpose of conducting the parameter sensitivity analysis experiment is to observe the changes in algorithm performance by adjusting the value of the scaling factor *β*, and thereby select the most suitable parameter value. In the code provided, *β* controls the update rate of parameter *a*, which determines the shape of the Sigmoid function. This parameter affects the rate at which *a* decreases from 2 to 0, directly controlling the balance between exploration and exploitation in the iterative process of the algorithm. The larger the value of *β*, the more abrupt the change of *a* from 2 to 0. Conversely, the smaller the value of *β*, the smoother the change of *a*. The main significance of this experiment is to optimize the performance of the algorithm, enhance its adaptability, and ensure that it performs well across different optimization problems. By adjusting *β*, the algorithm’s balance between global search in the early stages and local search in the later stages can be better controlled, thereby affecting the final optimization outcome. In this experiment, *β* values of 10, 15, 20, and 25 were selected for testing. The number of iterations, *T*, was set to 500, and the population size, *N*, was set to 30. Each algorithm was run 30 times individually on 23 classical benchmark functions for performance analysis. The Friedman values of the algorithms were recorded. As shown in Table 4, the RWOA with *β* = 25 achieved the smallest average Friedman value and ranked first. Therefore, *β* = 25 was chosen for this study.

**Table 4:** Friedman values of the RWOAs.

Metrics	RWOA (*β*=10)	RWOA (*β*=15)	RWOA (*β*=20)	RWOA (*β*=25)
Average Friedman Value	3.0761	2.7207	2.3158	1.8875
Rank	4	3	2	1

### 7.3 Qualitative analysis experiment

**Fig 9:**
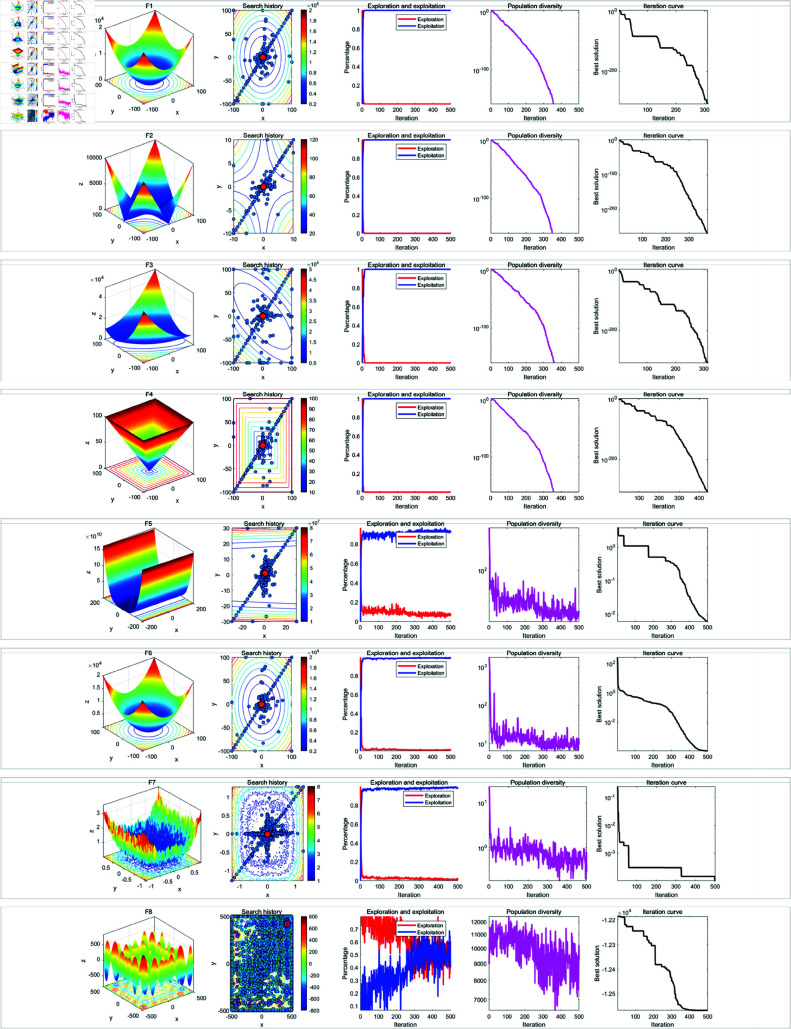
Results of qualitative analysis experiment (F1-F8).

**Fig 10:**
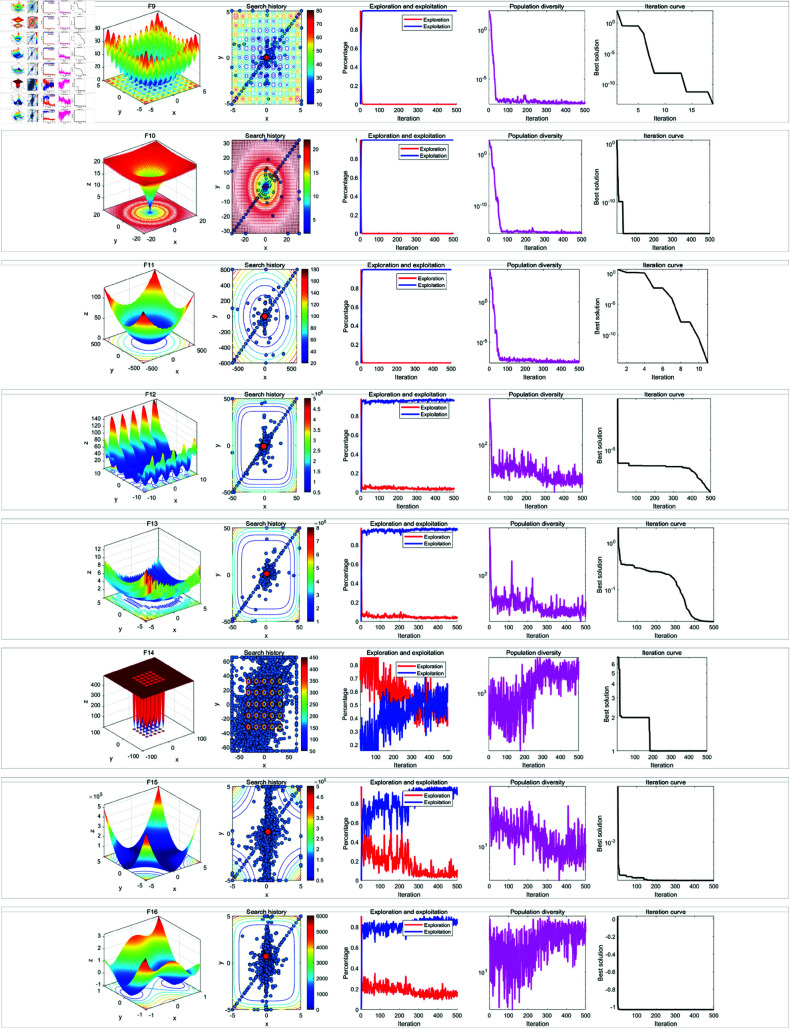
Results of qualitative analysis experiment (F1-F8).

**Fig 11:**
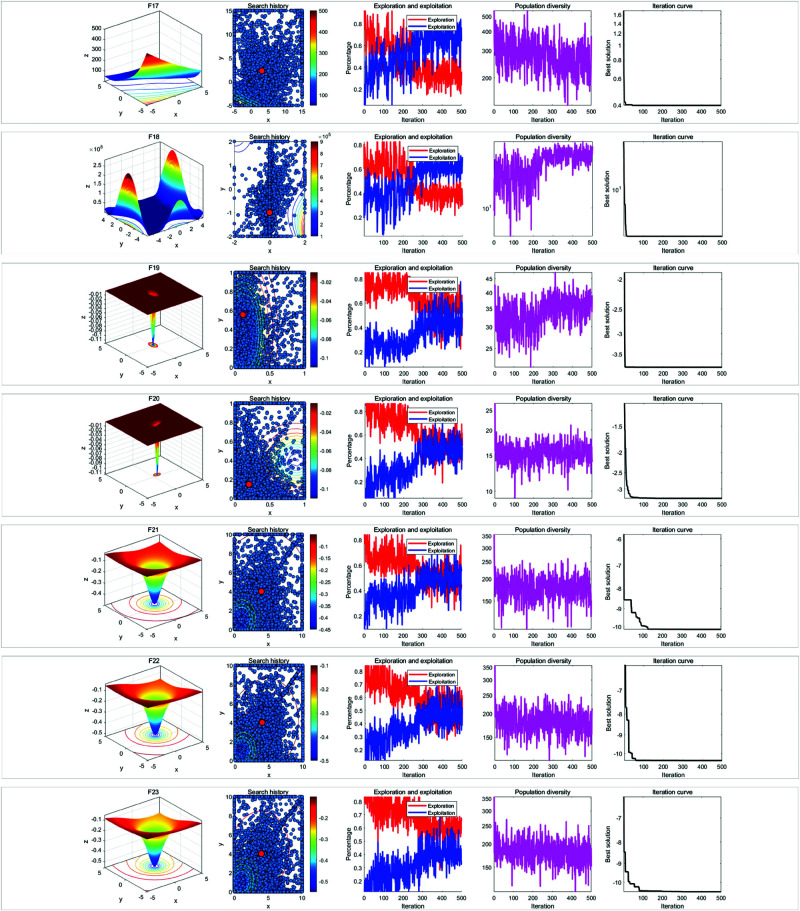
Results of qualitative analysis experiment (F1-F8).

In the qualitative analysis experiment, we set the number of iterations to *T* = 500 and the population size to *N* = 30, and ran RWOA independently on 23 benchmark functions in Table 2 to analyze the search history, exploration-exploitation ratio, and population diversity of RWOA. In addition, we provided the landscape of the benchmark functions and the iteration curves for reference. The results of the qualitative analysis were shown in Fig 9, Fig 10 and Fig 11, which includes:

- Landscapes of benchmark functions;
- Search history of the whale population;
- Exploration-exploitation ratio;
- Population diversity curves;
- Iteration curves.

The search history represents the positions and distribution of the whale individuals. In the search history graph of the whale population, the red circles indicate the global optimum position, while the blue circles represent the search history of the whale individuals. Notably, RWOA effectively explored the entire search space. In single-modal functions such as F1-F4, RWOA exhibited fast convergence, with whale individuals finding the optimal solution within a limited number of iterations, leading to a concentrated distribution of individuals in the solution space. In the case of complex multi-modal functions like F8 and F17-F23, where many local optima exist, RWOA first performed rapid global exploration and then refined its search in promising regions. As a result, the whale individuals traversed a large portion of the solution space, and the search history was mainly concentrated around the optimal solution. In terms of balancing exploration and exploitation, RWOA performed excellently, effectively managing this trade-off. When dealing with functions like F1-F4 and F9-F1, RWOA showed a rapid increase in the exploitation ratio during the early iterations, demonstrating its strong exploration capability. In the case of functions like F14-F23, RWOA exhibited a higher exploitation ratio in the early iterations, with a slow decline in the exploitation ratio. This highlights its robust global exploration ability and local exploitation capability. For functions such as F5-F8 and F14-F23, the diversity curve of RWOA’s population consistently fluctuated and maintains high values. This indicates that RWOA can maintain high population diversity when handling complex multi-modal functions, effectively preventing premature convergence caused by population clustering in certain areas.

### 7.4 Comparative experiment of different algorithms

**Fig 12:**
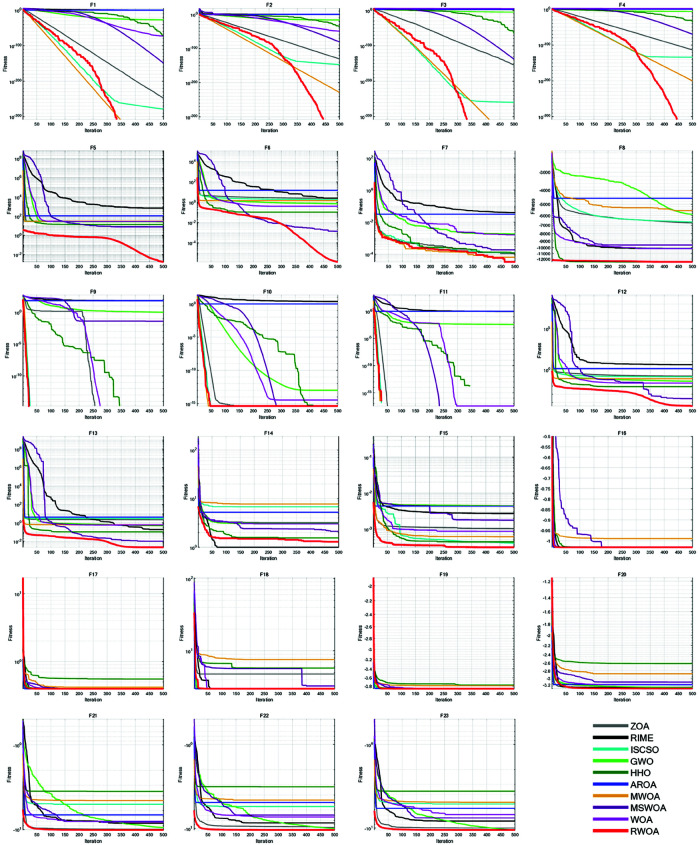
Iteration curves of different algorithms in comparison experiment.

To further validate the superiority of RWOA, we selected Zebra Optimization Algorithm (ZOA) [40], Rime Optimization Algorithm (RIME) [41], Improved Sand Cat Swarm Optimization Algorithm (ISCSO) [42], Grey Wolf Optimizer (GWO) [8], Harris Hawks Optimization (HHO) [4], Attraction-Repulsion Optimization Algorithm (AROA) [43], MWOA [44], MSWOA [45], and Whale Optimization Algorithm (WOA) [3] for comparison, and tested them on the benchmark functions listed in Table 2. The parameter settings for each algorithm are shown in Table 5. The number of iterations was uniformly set to *T*=500, and the population size to *N*=30. Each algorithm was independently run 30 times on 23 benchmark functions, and the average fitness (Ave), standard deviation (Std), *p*-values of the Wilcoxon rank-sum test, and Friedman values were recorded for performance analysis. The experimental results are shown in Fig 12, Tables 7 and 8.

**Table 5:** Parameter settings for algorithms.

Algorithm	Parameter(s)	Value
ZOA [40]	*R*	0.1
ISCSO [42]	Maximum Sensitivity Range *S*	2
	*R*	[-2, 2]
RIME [41]	*ω*	5
GWO [8]	Convergence Factor *a*	2 decreasing to 0
HHO [4]	Threshold	0.5
	Energy *E*	|2 |to 0
AROA [43]	Attraction factor *c*	0.95
	Local search scaling factor 1	0.15
	Local search scaling factor 2	0.6
	Attraction probability 1	0.2
	Local search probability	0.8
	Expansion factor	0.4
	Local search threshold 1	0.9
	Local search threshold 2	0.85
	Local search threshold 3	0.9
MWOA [44]	Convergence Factor *a*	2 decreasing to 0
	Spiral Factor *b*	1
	${CF}_{1}$	2.5
	${CF}_{2}$	2.5
MSWOA [45]	Convergence Factor *a*	2 decreasing to 0
	Spiral Factor *b*	1
	Inertia Weight *ω*	From 0.5 to 4.5
WOA [3]	Convergence Factor *a*	2 decreasing to 0
	Spiral Factor b	1
RWOA	Convergence Factor *a*	2 decreasing to 0
	Spiral Factor *b*	1
	Scaling Factor *β*	25

**Table 6:** Details of the metaheuristic algorithms.

Algorithm	Year	Author(s)	Source of Inspiration
Zebra Optimization Algorithm	2022	E Trojovská et al.	Foraging and defense
(ZOA) [40]			strategy of zebras.
Grey Wolf Optimizer (GWO) [8]	2014	Seyedali Mirjalili et al.	The leadership hierarchy and
			hunting system of gray wolves.
Rime optimization algorithm	2023	Su Hang et al.	The formation process of
(RIME) [41]			rime in nature.
Improved Sand Cat Swarm	2024	Ying Li et al.	Nonlinear convergence strategy,
Optimization algorithm (ISCSO) [42]			perturbation factor
Harris Hawk Optimization	2019	AA Heidari et al.	The predatory behavior of
algorithm (HHO) [4]			Harris hawks.
Attraction-Repulsion Optimization	2024	K Cymerys	Attraction-repulsion phenomenon.
Algorithm (AROA) [43]			
MWOA [44]	2021	Anitha J et al.	The cosine function,
			the correction factors.
MSWOA [45]	2022	Yang Wenbiao et al.	Adaptive inertia weight, dynamic
			convergence factor and Levy flight.
Whale Optimization Algorithm	2016	Seyedali Mirjalili et al.	The hunting behavior of
(WOA) [3]			humpback whales.

**Table 7:** Comparative results of each algorithm in comparative experiment.

Function	Metrics	ZOA	RIME	ISCSO	GWO	HHO	AROA	MWOA	MSWOA	WOA	RWOA
F1	Ave	7.2922E-249	2.1665E+00	2.7093E-287	1.5545E-27	9.6731E-72	5.2703E+00	0.0000E+00	1.0581E-149	4.8160E-74	0.0000E+00
	Std	7.7642E-249	8.0032E-01	3.3422E-287	2.2657E-27	5.1512E-71	4.4161E+00	0.0000E+00	2.3537E-149	1.7219E-73	0.0000E+00
F2	Ave	6.4623E-131	6.9900E-01	1.1487E-146	8.5308E-17	3.6246E-37	7.0830E-01	9.2690E-229	8.1498E-81	1.1281E-49	0.0000E+00
	Std	2.9716E-130	1.5181E+00	4.9604E-146	3.8864E-17	1.3396E-36	3.0046E-01	9.7640E-229	2.3685E-80	6.0079E-49	0.0000E+00
F3	Ave	1.5426E-157	1.2995E+03	1.3400E-259	7.6892E-06	5.2059E-62	2.1541E+02	0.0000E+00	3.6589E-138	4.5096E+04	0.0000E+00
	Std	6.2219E-157	3.9296E+02	2.5321E-259	1.2087E-05	2.8512E-61	2.7919E+02	0.0000E+00	8.9593E-138	1.3289E+04	0.0000E+00
F4	Ave	6.8370E-114	7.5693E+00	1.6778E-137	8.4292E-07	5.6568E-35	1.4868E+00	1.3702E-200	1.4090E-70	4.8940E+01	0.0000E+00
	Std	2.8896E-113	3.2049E+00	7.6689E-137	8.9549E-07	3.0265E-34	4.9530E-01	2.4630E-200	9.0126E-71	2.5144E+01	0.0000E+00
F5	Ave	2.8268E+01	6.9507E+02	2.7803E+01	2.7280E+01	1.3537E+01	1.0704E+02	2.8698E+01	7.7090E+00	2.7909E+01	1.7651E-03
	Std	5.2158E-01	9.0021E+02	8.0903E-01	8.3217E-01	1.3891E+01	9.2542E+01	1.1043E-01	1.2810E+01	3.9822E-01	2.6606E-03
F6	Ave	2.4540E+00	2.5001E+00	1.7774E+00	7.2068E-01	1.0503E-01	1.4566E+01	1.4031E+00	1.3707E-03	3.9984E-01	1.3559E-06
	Std	6.4148E-01	1.0475E+00	5.1706E-01	2.7752E-01	1.6055E-01	9.0777E+00	4.0197E-01	1.0986E-03	1.7710E-01	1.2529E-06
F7	Ave	1.0818E-04	3.9793E-02	1.2468E-04	1.8705E-03	1.2233E-04	3.1407E-02	6.5054E-05	1.8801E-04	1.6350E-03	3.3639E-05
	Std	5.5379E-05	1.4327E-02	1.0691E-04	8.5858E-04	1.1346E-04	3.9162E-02	7.2464E-05	1.0448E-04	1.2119E-03	2.9750E-05
F8	Ave	-6.7330E+03	-10142.4676	-6.6443E+03	-5880.9569	-1.2569E+04	-4.5483E+03	-5.5468E+03	-9.6159E+03	-10151.6855	-1.2569E+04
	Std	7.8507E+02	4.6656E+02	9.5133E+02	8.7087E+02	5.1504E-01	7.0120E+02	2.0385E+03	1.5313E+03	1.8180E+03	3.8796E-02
F9	Ave	0.0000E+00	5.9570E+01	0.0000E+00	3.1764E+00	0.0000E+00	5.3576E+01	0.0000E+00	4.6347E-02	1.8948E-15	0.0000E+00
	Std	0.0000E+00	1.2063E+01	0.0000E+00	4.3832E+00	0.0000E+00	7.1889E+01	0.0000E+00	2.5385E-01	1.0378E-14	0.0000E+00
F11	Ave	0.0000E+00	9.7587E-01	0.0000E+00	5.6127E-03	0.0000E+00	9.9602E-01	0.0000E+00	0.0000E+00	0.0000E+00	0.0000E+00
	Std	0.0000E+00	4.9164E-02	0.0000E+00	9.3749E-03	0.0000E+00	1.0981E-01	0.0000E+00	0.0000E+00	0.0000E+00	0.0000E+00
F12	Ave	0.16326	4.0169	0.13696	0.038153	0.0083798	1.2982E+00	7.5933E-02	2.8399E-04	2.2783E-02	3.6331E-05
	Std	7.4035E-02	1.6759E+00	5.6999E-02	1.4280E-02	1.0538E-02	3.0895E-01	3.4500E-02	3.0712E-04	1.1898E-02	8.8818E-05
F13	Ave	2.1804E+00	1.8836E-01	2.9226E+00	4.9487E-01	1.0290E-01	4.0851E+00	5.9967E-01	9.9820E-03	5.8808E-01	2.2077E-03
	Std	3.7582E-01	6.9286E-02	6.2417E-02	1.9866E-01	1.3109E-01	3.8920E-01	1.8873E-01	1.8587E-02	2.1944E-01	4.6310E-03
F14	Ave	2.0875E+00	9.9800E-01	6.6512E+00	2.6615E+00	2.4631E+00	5.0285E+00	9.4107E+00	1.9889E+00	2.2820E+00	1.0964E+00
	Std	1.4287E+00	2.9752E-12	5.6156E+00	3.6123E+00	3.6470E+00	3.4140E+00	4.3844E+00	1.6802E+00	2.0294E+00	6.2743E-01
F15	Ave	1.0318E-03	2.7247E-03	4.0471E-04	4.4293E-03	4.3840E-04	4.3788E-03	6.1215E-04	1.7709E-03	8.6749E-04	3.1045E-04
	Std	3.6568E-03	5.9863E-03	1.9508E-04	8.1062E-03	2.4903E-04	6.7853E-03	1.6444E-04	3.9878E-03	1.1906E-03	7.8859E-06
F16	Ave	-1.0316E+00	-1.0316E+00	-1.0316E+00	-1.0316E+00	-1.0316E+00	-1.0316E+00	-9.9164E-01	-1.0311E+00	-1.0316E+00	-1.0316E+00
	Std	6.5106E-09	1.0085E-07	8.3437E-09	2.4786E-08	4.7731E-07	3.0844E-05	3.7601E-02	2.5522E-03	1.3995E-09	1.0512E-15
F17	Ave	3.9789E-01	3.9789E-01	3.9789E-01	3.9789E-01	5.5283E-01	3.9840E-01	4.1824E-01	3.9814E-01	3.9789E-01	3.9789E-01
	Std	4.1800E-08	7.3421E-07	6.7385E-07	4.2251E-06	8.4751E-01	2.2266E-03	2.6777E-02	3.5820E-04	6.4496E-06	5.5652E-14
F18	Ave	4.8000E+00	3.0000E+00	3.0000E+00	3.0001E+00	5.7901E+00	3.0008E+00	7.6009E+00	3.2696E+00	3.0000E+00	3.0000E+00
	Std	6.8501E+00	1.5305E-06	8.8737E-06	7.1303E-05	8.5213E+00	1.7734E-03	9.0914E+00	1.4748E+00	7.7777E-04	9.5939E-07
F19	Ave	-3.8623E+00	-3.8628E+00	-3.8618E+00	-3.8617E+00	-3.7727E+00	-3.8557E+00	-3.7876E+00	-3.8604E+00	-3.8557E+00	-3.8628E+00
	Std	4.8455E-04	4.0676E-07	2.4118E-03	2.3790E-03	9.7508E-02	2.9506E-02	6.4787E-02	1.9904E-03	9.5302E-03	8.0059E-09
F20	Ave	-3.2831E+00	-3.2705E+00	-3.1976E+00	-3.2752E+00	-2.6112E+00	-3.1876E+00	-2.8769E+00	-3.1124E+00	-3.1887E+00	-3.3020E+00
	Std	5.2792E-02	5.9919E-02	2.0107E-01	6.3500E-02	5.1723E-01	5.5063E-02	2.2108E-01	3.7975E-02	1.9758E-01	1.4488E-02
F21	Ave	-9.6429E+00	-8.5493E+00	-5.1168E+00	-8.9767E+00	-3.2436E+00	-5.6180E+00	-4.5545E+00	-8.3620E+00	-7.3519E+00	-1.0153E+01
	Std	1.5554E+00	2.5261E+00	1.7289E+00	2.4460E+00	1.6660E+00	3.1884E+00	3.4328E-01	2.3814E+00	3.1168E+00	7.1450E-11
F22	Ave	-9.5164E+00	-9.1881E+00	-5.5416E+00	-1.0224E+01	-3.3689E+00	-6.2735E+00	-4.4841E+00	-7.2516E+00	-6.8815E+00	-1.0403E+01
	Std	2.0145E+00	2.5196E+00	1.7010E+00	9.7013E-01	1.6144E+00	3.0858E+00	8.1829E-01	3.3736E+00	3.1970E+00	5.5113E-11
F23	Ave	-9.6351E+00	-9.6987E+00	-6.0297E+00	-1.0264E+01	-3.3794E+00	-6.4335E+00	-4.6633E+00	-6.6241E+00	-7.4771E+00	-1.0536E+01
	Std	2.0499E+00	2.1981E+00	2.0496E+00	1.4812E+00	1.3646E+00	3.3003E+00	1.0740E+00	3.5638E+00	3.4247E+00	8.5124E-11

As can be seen from Fig 12, Tables 7 and 8, the average fitness (Ave) and standard deviation (Std) of RWOA are superior to other algorithms in F1-F13 and F15-F23. This proves that RWOA had better robustness, adaptability and stability than other algorithms when dealing with uni-modal problems or most complex multi-modal problems. In F1 and F3, both MWOA and RWOA found the optimal solution within a limited number of iterations. In F9-F11, the performance of both RWOA and ZOA, ISCSO, HHO, MWOA is the best, which also proves that RWOA had the ability to find the optimal solution to complex problems without losing to other algorithms. However, in F14, although RWOA had been optimized to the optimal solution for many times, its overall average fitness and standard deviation are slightly worse than RIME, indicating that RWOA was less stable than RIME when solving problems like F14, confirming the famous ’No free lunch (NFL) theorem’: RWOA is not perfect, it performs well in the vast majority of benchmark functions, but may perform slightly worse than individual algorithms on a few functions.

However, in the performance evaluation of optimization algorithm, the convergence and stability of the algorithm are usually measured by average fitness and standard deviation, which can not directly reflect the performance of the algorithm. Comparing the performance of different algorithms by average fitness and standard deviations alone has certain limitations, so non-parametric tests are often introduced: Wilcoxon rank sum tests and Friedman tests.

The Wilcoxon rank-sum test is a non-parametric test that is used to statistically test the difference between the medians of two independent samples. In the comparison of optimization algorithms, when we wish to compare whether there is a significant difference between the effects of two algorithms, Wilcoxon rank-sum test can help us determine whether the difference is statistically significant. If the *p*-value of Wilcoxon rank-sum test is less than a set of significance levels (typically 0.05), it can be assumed that the performance difference between the two algorithms is significant and not just due to random error. As can be seen from Table 8, RWOA had significant differences with RIME, GWO and MWOA in all benchmark functions.

The Friedman test is a non-parametric analysis of variance used to compare the performance of multiple algorithms on different test problems. It can identify if there are statistically significant differences between algorithms. Friedman test can effectively eliminate the bias between samples by comparing the performance of multiple algorithms in multiple data sets or test environments, providing a fairer comparison and avoiding the multiple comparison problem caused by comparing multiple algorithms individually. As shown in Table 8, the average Friedman value of RWOA is 1.6833, ranking first among the selected SOTA algorithms.

Table 9 summarizes all performance results of RWOA and other algorithms by a useful metric named overall effectiveness (OE). In Table 9, *w* indicates win, *t* indicates tie and *l* indicates loss. The OE of each algorithm is computed by Eq 33 [46].


$$\begin{array}{ccc} OE=\frac{N-L}{L}\cdot 100 & & \end{array}$$ (33)

where *N* is the total number of tests; *L* is the total number of losing tests for each algorithm.

**Table 8:** Result of non-parametric tests of different algorithms.

Algorithm	Average Friedman Value	Rank	(+/=/-)
ZOA	4.3638	2	20/3/0
RIME	6.5790	9	23/0/0
ISCSO	4.9942	3	20/3/0
GWO	5.8754	7	23/0/0
HHO	5.7043	5	20/3/0
AROA	8.3754	10	20/3/0
MWOA	6.2580	8	23/0/0
MSWOA	5.3235	4	18/5/0
WOA	5.8442	6	21/2/0
RWOA	**1.6833**	**1**	-

RWOA with overall effectiveness of 100.00% was the most effective algorithm. And RWOA was competitive with other SOTA algorithms on the benchmark functions. The results revealed the ability of RWOA to handle optimization problems. In summary, after comparing average fitness and standard deviation, *p*-values of Wilcoxon rank-sum test and Friedman values of Friedman test, RWOA performed best, which proved the superiority of RWOA.

## 8 Engineering design optimization

In engineering design optimization simulations, we employed the Penalty Function Method to handle the constraints of the optimization problem. The Penalty Function Method is a widely used and effective constraint handling technique. This approach transforms the constrained optimization problem into an unconstrained one by incorporating a *punishment* term into the objective function, simplifying the solution process. When a variable $x_{i}$ violates a constraint, the penalty function imposes a significant penalty, thereby guiding the algorithm to favor solutions that satisfy the constraints.

A comparative study was conducted between RWOA and other algorithms including Zebra Optimization Algorithm (ZOA) [40], Rime Optimization Algorithm (RIME) [41], Improved Sand Cat Swarm Optimization Algorithm (ISCSO) [42], Grey Wolf Optimizer (GWO) [8], Harris Hawks Optimization (HHO) [4], Attraction-Repulsion Optimization Algorithm (AROA) [43], MWOA [44], MSWOA [45] and Whale Optimization Algorithm (WOA) [3]. Parameter settings for each algorithm are shown in Table 5, with the maximum number of iterations *T*=500 and population size *N*=30 uniformly. Each algorithm was run independently 30 times, recording the average fitness value (Ave) and standard deviation (Std) for performance analysis. The experimental results were presented in Figs 14, 16, 18, 20, 22, 24, 26, 28, 30 and Table 10.

**Table 9:** Effectiveness of RWOA and other SOTA algorithms.

Metrics	ZOA	RIME	ISCSO	GWO	HHO	AROA	MWOA	MSWOA	WOA	RWOA
	(*w*/*t*/*l*)	(*w*/*t*/*l*)	(*w*/*t*/*l*)	(*w*/*t*/*l*)	(*w*/*t*/*l*)	(*w*/*t*/*l*)	(*w*/*t*/*l*)	(*w*/*t*/*l*)	(*w*/*t*/*l*)	(*w*/*t*/*l*)
Total	0/3/20	0/0/23	0/3/20	0/0/23	0/3/20	0/3/20	0/0/23	0/5/18	0/2/21	18/5/0
*OE*	13.04%	0.00%	13.04%	0.00%	13.04%	13.04%	0.00%	21.74%	8.70%	**100.00%**

**Table 10:** Average fitness and standard deviation of each algorithm across the seven engineering design problems.

Challenges	Metrics	ZOA	RIME	ISCSO	GWO	HHO	AROA	MWOA	MSWOA	WOA	RWOA
Three-bar Truss	Ave	259.805047	259.806360	259.805060	259.805060	259.814636	259.823541	260.039940	259.808306	259.879210	**259.805047**
	Std	0.000003	0.001404	0.000025	0.000013	0.023965	0.049850	0.239266	0.006063	0.098045	**0.000000**
Tension/Compression Spring	Ave	0.121523	0.124562	0.121524	0.121525	0.121525	0.130463	11.740844	0.121536	0.121726	**0.121522**
	Std	0.000001	0.015331	0.000002	0.000004	0.000016	0.018277	44.455569	0.000014	0.001113	**0.000000**
Speed Reducer	Ave	2638.821923	2638.900901	2638.874170	2638.847608	2638.972627	2639.875439	2716.300093	2639.131376	2639.509715	**2638.819857**
	Std	0.004772	0.138287	0.074977	0.023036	0.832107	1.755889	40.940720	0.625069	3.778187	**0.000094**
Cantilever Beam	Ave	13.360278	13.641036	13.360834	13.360447	13.391244	20.85846	14.125428	13.414989	15.761032	**13.360259**
	Std	0.000026	0.275719	0.000531	0.000161	0.021703	5.466751	0.461674	0.039764	1.642067	**0.000000**
Pressure Vessel	Ave	1527.696092	1375.194872	1167.676729	1115.959892	1440.725403	1442.454141	5864.296095	1223.175940	1329.818716	**1115.909538**
	Std	529.085175	232.654406	196.940627	0.042239	309.711668	290.764311	2652.934293	325.771679	316.756207	**0.000009**
I-beam	Ave	6.702966	6.506251	6.702891	6.702760	6.702776	5.987401	5.261520	6.697431	6.057699	**6.703048**
	Std	0.000136	0.199303	0.000142	0.000321	0.000512	0.866697	1.291070	0.006149	0.571504	**0.000000**
Piston lever	Ave	8.933115	65.486147	17.749454	17.736635	251.219834	268.815025	226.897883	12.784060	46.653239	**1.057175**
	Std	33.860122	112.043588	50.930710	50.885577	214.502634	155.003223	178.445211	44.491746	84.577607	**0.000000**
Reactor Network	Ave	0.343214	0.320908	0.183703	0.075062	2.596082	6.483337	34.455371	0.041646	1.312730	**0.000000**
	Std	0.023196	0.059702	0.187679	0.102938	3.735463	8.416040	39.401636	0.200375	1.739066	**0.000000**
Gas Transmission System	Ave	1224745.938257	1224745.981102	1224745.953883	1224745.964832	1224745.937224	1226144.165641	1293659.097070	1224748.701193	1224745.937230	**1224745.937222**
	Std	0.002312	0.096923	0.024127	0.025926	0.000009	4283.591039	29208.999580	5.093276	0.000025	**0.000000**

### 8.1 Three-bar truss

**Fig 13:**
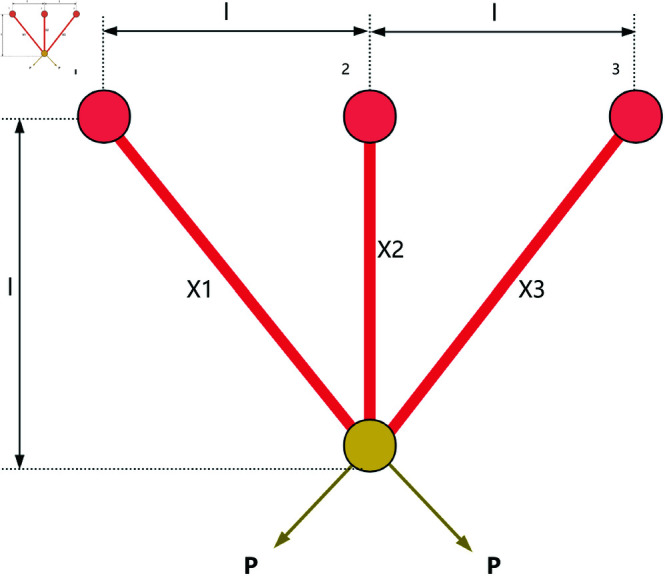
The structure of a three-bar truss.

The three-bar truss is a simple structural system consisting of three members, as shown in Fig 13. It is commonly used to support concentrated loads and is widely applied in engineering fields such as bridges, buildings, and aerospace. The three-bar truss design problem is a classic structural optimization problem, often used to study the mechanical behavior of simple structures under external loading conditions. In the three-bar truss design problem, the objective is to optimize the cross-sectional areas of the truss members to minimize material usage while ensuring that the structure meets the required mechanical performance.

This optimization problem involves a nonlinear objective function, three nonlinear inequality constraints, and two continuous decision variables $x_{1}$ and $x_{2}$. The objective function for the three-bar truss design problem can be described as follows:

*Variable:*


$$\begin{array}{lll} \begin{array}{c} x=[x_{1},x_{2}] \end{array} & \; & \end{array}$$

*Minimize:*


$$\begin{array}{ccc} f(x)=(2\sqrt{2}x_{1}+x_{2})\cdot l+punishment & & \end{array}$$ (34)

*Subject to:*


$$\begin{array}{c} g_{1}(x)=\frac{\sqrt{2}x_{1}+x_{2}}{\sqrt{2}\cdot x_{1}^{2}+2x_{1}\cdot x_{2}}P-\sigma \leq 0 \end{array}$$ (35)

$$\begin{array}{c} g_{2}(x)=\frac{x_{2}}{\sqrt{2}x_{1}^{2}+2x_{1}\cdot x_{2}}P-\sigma \leq 0 \end{array}$$ (36)

$$\begin{array}{c} g_{3}(x)=\frac{1}{\sqrt{2}x_{2}+x_{1}}P-\sigma \leq 0 \end{array}$$ (37)

*Where:*


$$\begin{array}{lll} \begin{array}{c} l=100cm;\;P=2kN∕cm^{2};\;\sigma =2kN∕cm^{2} \end{array} & \; & \end{array}$$

punishment=103⋅∑i=13 max ⁡ (0,g(i))2

*Variable range:*


$$\begin{array}{lll} \begin{array}{c} 0\leq x_{1}\leq 1,\;0\leq x_{2}\leq 2; \end{array} & \; & \end{array}$$

**Fig 14:**
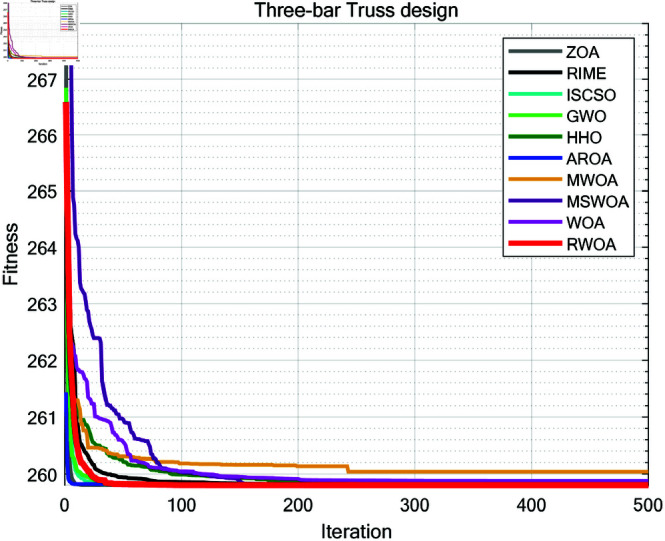
Iteration curves of the algorithms in three-bar truss design.

The experimental results are shown in Fig 14 and Table 10. From Table 10, it can be observed that RWOA significantly outperformed other algorithms in terms of stability, with the best optimization accuracy among all algorithms. This indicates that RWOA had a significant advantage when handling such optimization problems.

### 8.2 Tension/compression spring

**Fig 15:**
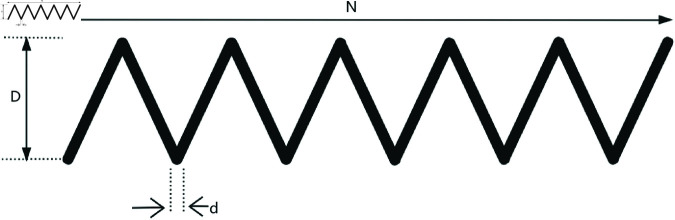
The structure of a tension/compression spring.

The extension/compression spring, as shown in Fig 15, plays a crucial role in modern industry, with widespread applications in fields such as automotive, home appliances, and electronics. Its design optimization not only helps improve product performance and extend service life, but also reduces costs and enhances manufacturing efficiency. Through reasonable design optimization, the spring can achieve optimal performance in dynamic working environments and meet various stringent requirements. The optimization objective of the design problem for the extension/compression spring is the minimization of its mass. The problem needs to be solved under constraints such as shear force, deflection, fluctuation frequency, and outer diameter. There are three design variables in this problem: coil diameter *d*, mean coil diameter *D*, and number of coils *N*. There are also four constraints, $g_{1}$ to $g_{4}$. The mathematical model of the problem is as follows,

*Variable:*


$$\begin{array}{lll} \begin{array}{c} x=[d,D,N]=[x_{1},x_{2},x_{3}] \end{array} & \; & \end{array}$$

*Minimize:*


$$\begin{array}{ccc} f(x)=(x_{3}+2)\cdot x_{2}\cdot x_{1}^{2}+punishment & & \end{array}$$ (38)

*Subject to:*


$$\begin{array}{c} g_{1}(x)=1-\frac{x_{2}^{3}\cdot x_{3}}{71785x_{1}^{4}}\leq 0 \end{array}$$ (39)

$$\begin{array}{c} g_{2}(x)=\frac{4x_{2}^{2}-x_{1}\cdot x_{2}}{12566(x_{2}\cdot x_{1}^{3}-x_{4})}+\frac{1}{5108x_{1}^{2}}-1\leq 0 \end{array}$$ (40)

$$\begin{array}{c} g_{3}(x)=1-\frac{140.45x_{1}}{x_{2}^{2}\cdot x_{3}}\leq 0 \end{array}$$ (41)

$$\begin{array}{c} g_{4}(x)=\frac{x_{1}+x_{2}}{1.5}-1\leq 0 \end{array}$$ (42)

*Where:*


punishment=103⋅∑i=14 max ⁡ (0,g(i))2

*Variable range:*


$$\begin{array}{lll} \begin{array}{c} 0.05\leq x_{1}\leq 2,\;0.25\leq x_{2}\leq 1.3,\;2.0\leq x_{3}\leq 15 \end{array} & \; & \end{array}$$

**Fig 16:**
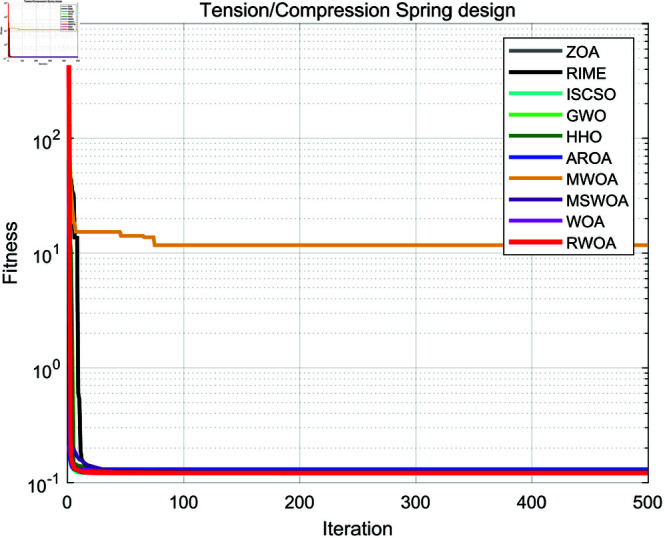
Iteration curves of the algorithms in tension/compression spring design.

The experimental results are presented in Fig 16, and Table 10. As shown in Table 10, the stability of RWOA in the tension/compression spring design problem significantly surpassed other algorithms, and it achieved the highest optimization accuracy among all algorithms. This indicates that RWOA had a significant advantage in handling such optimization problems.

### 8.3 Speed reducer

**Fig 17:**
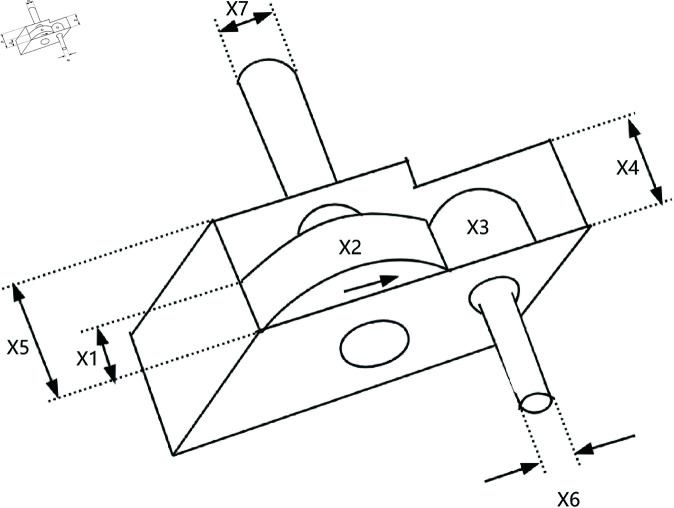
The structure of a speed reducer.

A speed reducer is a mechanical transmission device and one of the key components of a gearbox, shown in Fig 17. It is primarily used to reduce the rotational speed of an electric motor or other power sources while increasing the output torque. The reducer achieves this speed reduction through gears, worm gears, or other transmission mechanisms. It is typically applied in situations where there is a need to decrease the rotational speed, increase torque, or adjust the direction of motion.

In the optimization design of a reducer, the goal is to minimize the weight of the reducer. This problem involves seven variables, which are as follows: the width of the gear teeth $x_{1}$, the gear module $x_{2}$, the number of teeth on the small gear $x_{3}$, the length of the first shaft between the bearings $x_{4}$, the length of the second shaft between the bearings $x_{5}$, the diameter of the first shaft $x_{6}$, and the diameter of the second shaft $x_{7}$. Furthermore, this problem also involves eleven constraints, $g_{1}$ to $g_{1}1$. The mathematical formulation of the problem is as follows,

*Variable:*


$$\begin{array}{lll} \begin{array}{c} x=[x_{1},x_{2},x_{3},x_{4},x_{5},x_{6},x_{7}] \end{array} & \; & \end{array}$$

*Minimize:*


$$\begin{array}{ccc} y=f(x)+punishment & & \end{array}$$ (43)

*Subject to:*


$$\begin{array}{c} g_{1}=\frac{27}{x_{1}\cdot x_{2}^{2}\cdot x_{3}}-1\leq 0; \end{array}$$ (44)

$$\begin{array}{c} g_{2}=\frac{397.5}{x_{1}\cdot x_{2}^{2}\cdot x_{3}^{2}}-1\leq 0; \end{array}$$ (45)

$$\begin{array}{c} g_{3}=\frac{1.93x_{4}^{3}}{x_{2}\cdot x_{6}^{4}\cdot x_{3}}-1\leq 0; \end{array}$$ (46)

$$\begin{array}{c} g_{4}=\frac{1.93x_{5}^{3}}{x_{2}\cdot x_{7}^{4}\cdot x_{3}}-1\leq 0; \end{array}$$ (47)

$$\begin{array}{c} g_{5}=\frac{\sqrt{16.91\cdot 10^{6}+{\left(\frac{745x_{4}}{x_{2}\cdot x_{3}}\right)}^{2}}}{110x_{6}^{3}}-1\leq 0; \end{array}$$ (48)

$$\begin{array}{c} g_{6}=\frac{\sqrt{157.5\cdot 10^{6}+{\left(\frac{745x_{4}}{x_{2}\cdot x_{3}}\right)}^{2}}}{85x_{7}^{3}}-1\leq 0; \end{array}$$ (49)

$$\begin{array}{c} g_{7}=\frac{x_{2}\cdot x_{3}}{40}-1\leq 0; \end{array}$$ (50)

$$\begin{array}{c} g_{8}=\frac{5x_{2}}{x_{1}}-1\leq 0; \end{array}$$ (51)

$$\begin{array}{c} g_{9}=\frac{x_{1}}{12x_{2}}-1\leq 0; \end{array}$$ (52)

$$\begin{array}{c} g_{10}=\frac{1.5x_{6}+1.9}{x_{4}}-1\leq 0; \end{array}$$ (53)

$$\begin{array}{c} g_{11}=\frac{1.1x_{7}+1.9}{x_{5}}-1\leq 0; \end{array}$$ (54)

*Where:*


punishment=103⋅∑i=111 max ⁡ (0,gi(x))2

*Variable range:*


$$\begin{array}{lll} 2.6\leq x_{1}\leq 3.6;\;0.7\leq x_{2}\leq 0.8;\;17\leq x_{3}\leq 28;\;7.3\leq x_{4}\leq 8.3; & \; & \\ 7.3\leq x_{5}\leq 8.3;\;2.9\leq x_{6}\leq 3.9;\;5\leq x_{7}\leq 5.5; & \; & \end{array}$$

**Fig 18:**
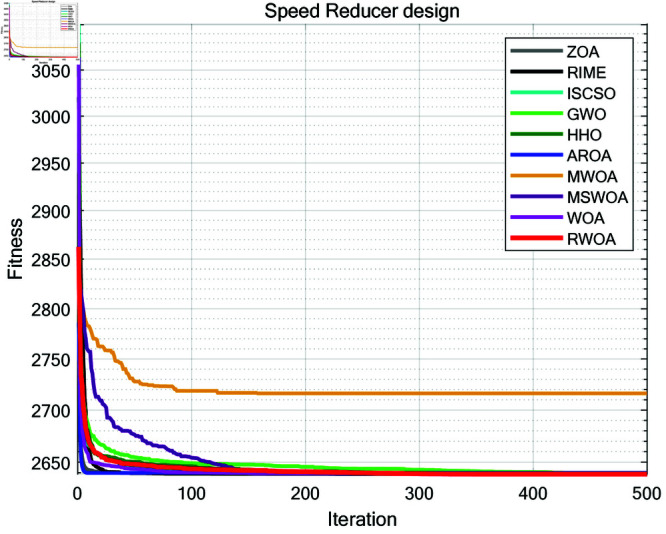
Iteration curves of the algorithms in speed reducer design.

The experimental results are presented in Fig 18, and Table 10. As shown in Table 10, the stability of RWOA in the Speed Reducer design problem significantly surpassed other algorithms, and it achieved the highest optimization accuracy among all algorithms. This indicates that RWOA had a significant advantage in handling such optimization problems.

### 8.4 Cantilever beam

**Fig 19:**
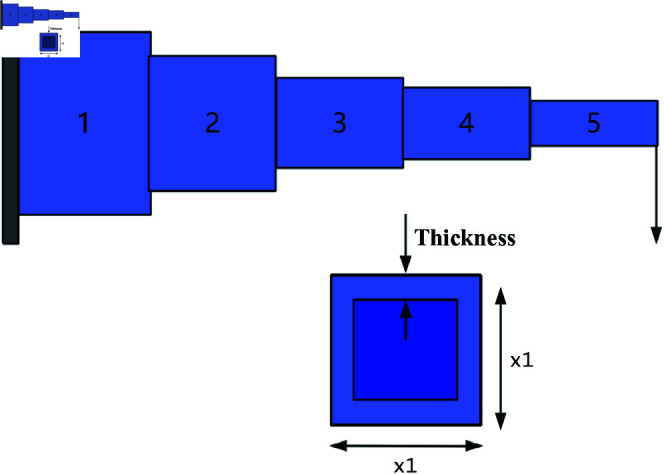
The structure of a cantilever beam.

A cantilever beam is a common structural form, fixed at one end and free at the other, as shown in Fig 19. The cantilever beam design problem is a classic engineering structural optimization problem, with the objective of minimizing material usage or beam weight while satisfying constraints on strength, stability, and other factors. This optimization problem is widely used in civil engineering, mechanical design, and aerospace fields.

The cantilever beam consists of five hollow square cross-section units. As shown in Fig 19, each unit is defined by one variable, and the thickness is constant. Therefore, the design problem includes five structural parameters, which correspond to five decision variables, denoted as $s_{1}$, $s_{2}$, $s_{3}$, $s_{4}$, $s_{5}$. The objective function for the cantilever beam design problem can be expressed as:

*Variable:*


$$\begin{array}{lll} \begin{array}{c} x=[s_{1},s_{2},s_{3},s_{4},s_{5}]=[x_{1},x_{2},x_{3},x_{4},x_{5}] \end{array} & \; & \end{array}$$

*Minimize:*


$$\begin{array}{ccc} f(x)=0.0624(x_{1}+x_{2}+x_{3}+x_{4}+x_{5})+punishment & & \end{array}$$ (55)

*Subject to:*


$$\begin{array}{ccc} g(x)=\frac{61}{x_{1}^{3}}+\frac{37}{x_{2}^{3}}+\frac{19}{x_{3}^{3}}+\frac{7}{x_{4}^{3}}+\frac{1}{x_{5}^{3}}-1\leq 0 & & \end{array}$$ (56)

*Where:*


$$\begin{array}{lll} \begin{array}{c} punishment=10^{3}\cdot \max{\left(0,g_{i}(x)\right)}^{2} \end{array} & \; & \end{array}$$

*Variable range:*


$$\begin{array}{lll} \begin{array}{c} 0.01\leq x_{i}\leq 100,\;i=1,2,3,4,5 \end{array} & \; & \end{array}$$

**Fig 20:**
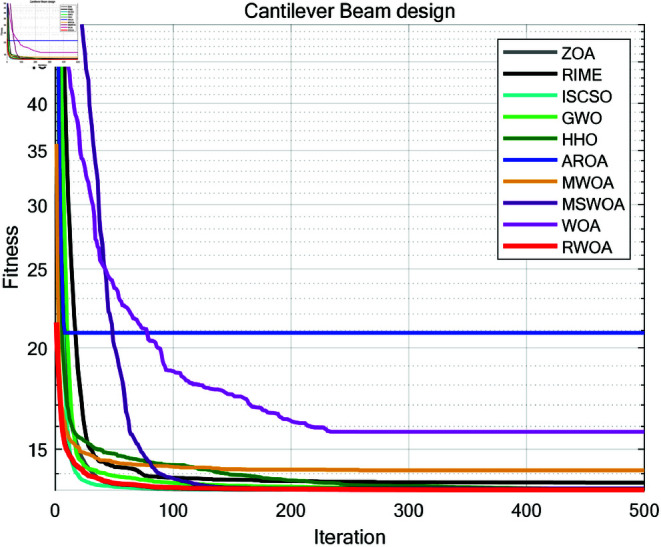
Iteration curves of the algorithms in cantilever beam design.

The experimental results are presented in Fig 20 and Table 10. As shown in Table 10, the stability of RWOA in the Cantilever Beam design problem significantly surpassed other algorithms, and it achieved the highest optimization accuracy among all algorithms. This indicates that RWOA had a significant advantage in handling such optimization problems.

### 8.5 Pressure vessel

**Fig 21:**
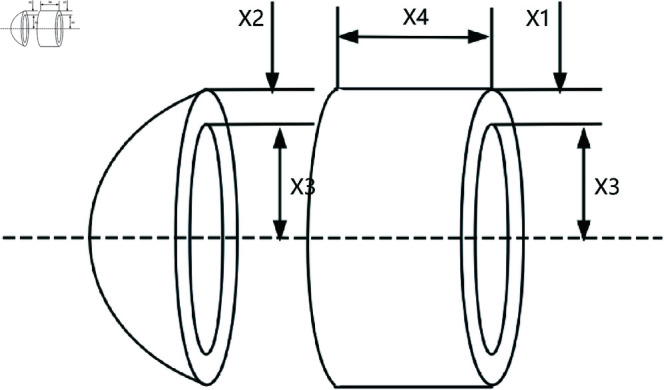
The structure of a pressure vessel.

A pressure vessel is a common mechanical structure used in fields such as chemical engineering, aerospace, and medical applications. The pressure vessel design problem is a classic structural optimization problem, where the goal is to minimize the manufacturing costs of the pressure vessel, including pairing, forming, and welding processes. The design of the pressure vessel is shown in Fig 21, with caps sealing both ends of the vessel. The cap at one end is hemispherical. $x_{1}$ and $x_{2}$ represent the wall thickness of the cylindrical section and the head, respectively, while $x_{3}$ is the inner diameter of the cylindrical section, and $x_{4}$ is the length of the cylindrical section, excluding the head. Thus, $x_{1}$, $x_{2}$, $x_{3}$, and $x_{4}$ are the four optimization variables of the pressure vessel design problem. The objective function and four optimization constraints are represented as follows:

*Variable:*


$$\begin{array}{lll} \begin{array}{cc} x & =[x_{1},x_{2},x_{3},x_{4}] \end{array} & \; & \end{array}$$

*Minimize:*


$$\begin{array}{ccc} f(x)=0.6224x_{1}\cdot x_{3}\cdot x_{4}+1.7781x_{2}\cdot x_{3}^{2}+3.1661x_{1}^{2}\cdot x_{4}+19.84x_{1}^{2}\cdot x_{3}+punishment & & \end{array}$$ (57)

*Subject to:*


$$\begin{array}{c} g_{1}(x)=-x_{1}+0.0193x_{3}\leq 0; \end{array}$$ (58)

$$\begin{array}{c} g_{2}(x)=-x_{2}+0.00954x_{3}\leq 0; \end{array}$$ (59)

$$\begin{array}{c} g_{3}(x)=-\pi x_{3}^{2}\cdot x_{4}-\frac{4}{3}\pi x_{3}^{2}+1296000\leq 0; \end{array}$$ (60)

$$\begin{array}{c} g_{4}(x)=x_{4}-240\leq 0; \end{array}$$ (61)

*Where:*


punishment=103⋅∑i=14 max ⁡ (0,gi(x))2

*Variable range:*


$$\begin{array}{lll} 0\leq x_{1}\leq 99;\;0\leq x_{2}\leq 99;\;10\leq x_{3}\leq 200;\;10\leq x_{4}\leq 99; & \; & \end{array}$$

**Fig 22:**
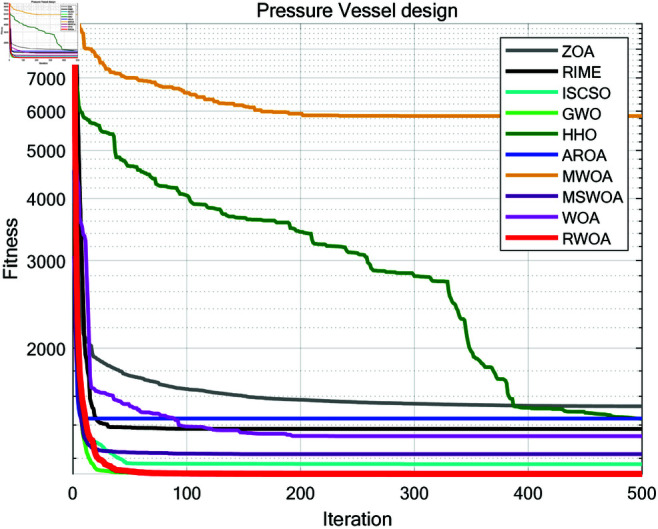
Iteration curves of the algorithms in pressure vessel design.

The experimental results are presented in Fig 22 and Table 10. As shown in Table 10, the stability of RWOA in the Pressure Vessel design problem significantly surpassed other algorithms, and it achieved the highest optimization accuracy among all algorithms. This indicates that RWOA had a significant advantage in handling such optimization problems.

### 8.6 I-beam

**Fig 23:**
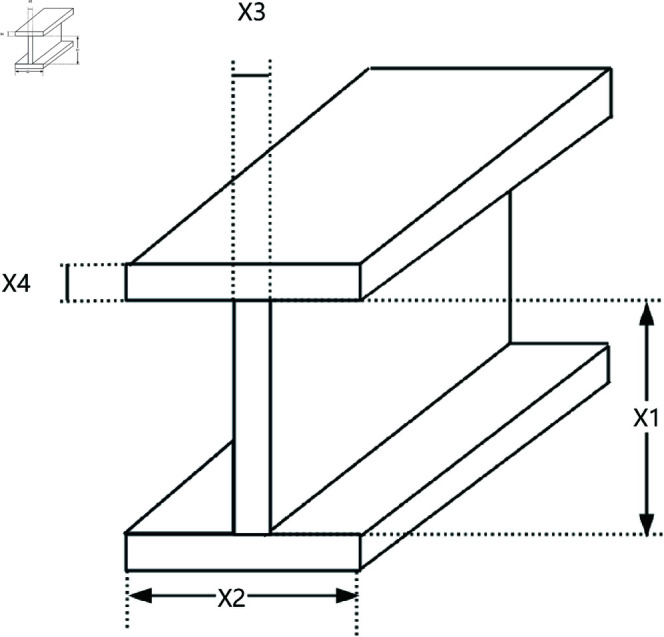
The structure of an I-beam.

An I-beam, named for its cross-sectional shape resembling the letter *I*, is a type of steel with high strength and low self-weight. It is widely used in various engineering structures. Its superior mechanical properties make it applicable in multiple fields, particularly in structures subjected to bending moments and axial forces. The objective of I-beam design optimization is to select the geometric parameters of the I-beam (such as width, height, thickness, etc.) in a way that maximizes its performance. This typically involves maximizing its load-bearing capacity, minimizing material usage, controlling structural deformations, and reducing costs. Optimizing I-beam design in engineering can enhance the safety, economy, and efficiency of structures. As shown in Fig 23, the I-beam design optimization problem involves four variables ($x_{1}$, $x_{2}$, $x_{3}$ and $x_{4}$) and two constraints ($g_{1}$ and $g_{2}$). $x_{1}$, $x_{2}$, $x_{3}$ and $x_{4}$ represent the web height, flange width, web thickness, and flange thickness of the I-beam, respectively. The objective function for the I-beam design problem can be described as:

*Variable:*


$$\begin{array}{lll} \begin{array}{c} x=[x_{1},x_{2},x_{3},x_{4}] \end{array} & \; & \end{array}$$

*Maximize:*


$$\begin{array}{ccc} f(x)=\frac{5000}{\frac{x_{3}\cdot {\left(x_{1}-2x_{4}\right)}^{3}}{12}+\frac{x_{2}\cdot x_{4}^{3}}{6}+2x_{2}\cdot x_{4}{\left(\frac{x_{1}-x_{4}}{2}\right)}^{2}}+punishment & & \end{array}$$ (62)

*Subject to:*


$$\begin{array}{c} g_{1}(x)=2x_{2}\cdot x_{3}+x_{3}\cdot (x_{1}-2x_{4})-300\leq 0; \end{array}$$ (63)

$$\begin{array}{c} g_{2}(x)=\frac{18\cdot 10^{4}x_{1}}{x_{3}{\left(x_{1}-2x_{4}\right)}^{3}+2x_{2}\cdot x_{3}\left(4x_{4}^{2}+3x_{1}\cdot (x_{1}-2x_{4})\right)}\leq 0 \end{array}$$ (64)

*Where:*


punishment=103⋅∑i=12 max ⁡ (0,gi(x))2

*Variable range:*


$$\begin{array}{lll} 10\leq x_{1}\leq 80;\;10\leq x_{2}\leq 50;\;0.9\leq x_{3}\leq 5;\;0.9\leq x_{4}\leq 5; & \; & \end{array}$$

**Fig 24:**
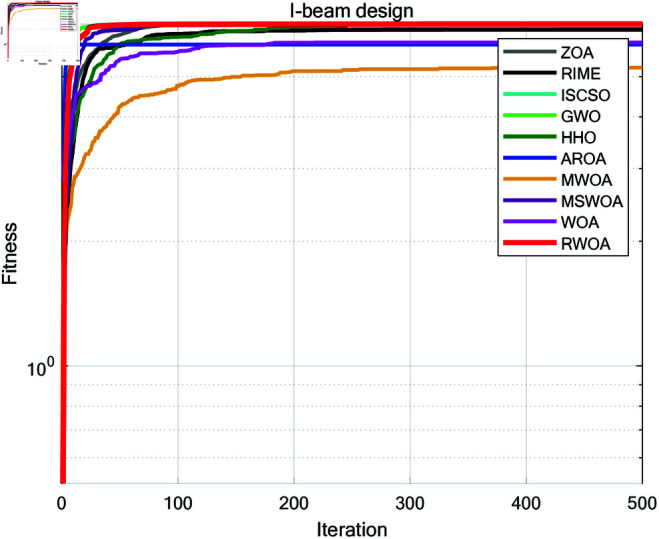
Iteration curves of the algorithms in I-beam design.

The experimental results are shown in Fig 24 and Table 10. As shown in Table 10, RWOA significantly outperformed the other algorithms in terms of both optimization accuracy and stability for the I-beam design problem. This demonstrates that RWOA had superior solving capabilities when handling this type of problem. This indicates that RWOA had a significant advantage in handling such optimization problems.

### 8.7 Piston lever

**Fig 25:**
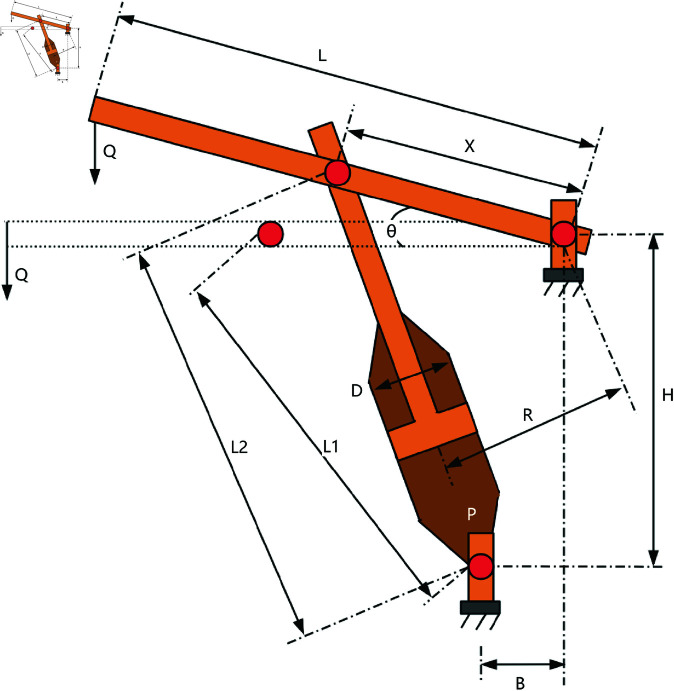
The structure of a piston lever.

In a factory, piston levers are critical components in various important pieces of equipment, such as pumps and compressors, where their performance directly impacts the reliability and efficiency of the entire system. Fig 25 is the structure of a piston lever. The piston lever design problem focuses on determining the optimal piston dimensions and material selection to ensure maximum performance in transmission systems. This design influences the efficiency, stability, and durability of mechanical systems. The optimization task seeks to balance dimensions, weight, material choice, and manufacturing cost in order to find the optimal piston size and material that maximize system efficiency and cost-effectiveness. Therefore, the piston lever design problem is of paramount importance in factories, and optimizing these key components is essential to ensuring the optimal performance of the system. The optimization involves four variables: piston length $x_{1}$, piston diameter $x_{2}$, material property $x_{3}$, and transmission rod length $x_{4}$, which affect the system’s mechanical properties, dynamic performance, and cost. The piston lever design problem can be described as:

*Variable:*


$$\begin{array}{lll} x=[x_{1},x_{2},x_{3},x_{4}] & \; & \end{array}$$

*Minimize:*


$$\begin{array}{ccc} f(x)=0.25\pi x_{3}^{2}(L_{2}-L_{1})+punishment & & \end{array}$$ (65)

*Subject to:*


g1(x)=QLcos ⁡ θ−RF≤0; (66)

$$\begin{array}{c} g_{2}(x)=Q(L-x_{4})-M_{\max}\leq 0; \end{array}$$ (67)

$$\begin{array}{c} g_{3}(x)=1.2(L_{2}-L_{1})-L_{1}\leq 0; \end{array}$$ (68)

$$\begin{array}{c} g_{4}(x)=\frac{x_{3}}{2}-x_{2}\leq 0; \end{array}$$ (69)

*Variable range:*


$$\begin{array}{lll} 0.05\leq x_{1}\leq 500;\;0.05\leq x_{2}\leq 500;\;0.05\leq x_{4}\leq 500;\;0.05\leq x_{3}\leq 120; & \; & \end{array}$$

*Where:*


$$\begin{array}{lll} Q=10000;\;P=1500;\;L=240;\;M_{\max}=1.8\times 10^{6}; & \; & \end{array}$$

L1=(x4−x2)2+x12;L2=(x4 sin ⁡ θ+x1)2+(x2−x4 cos ⁡ θ)2;

R=|−x4(x4 sin ⁡ θ+x1)+x1(x2−x4 cos ⁡ θ)|(x4−x2)2+x12;

$$\begin{array}{lll} F=0.25\pi Px_{3}^{2}; & \; & \end{array}$$

punishment=103⋅∑i=14 max ⁡ (0,gi(x))2

**Fig 26:**
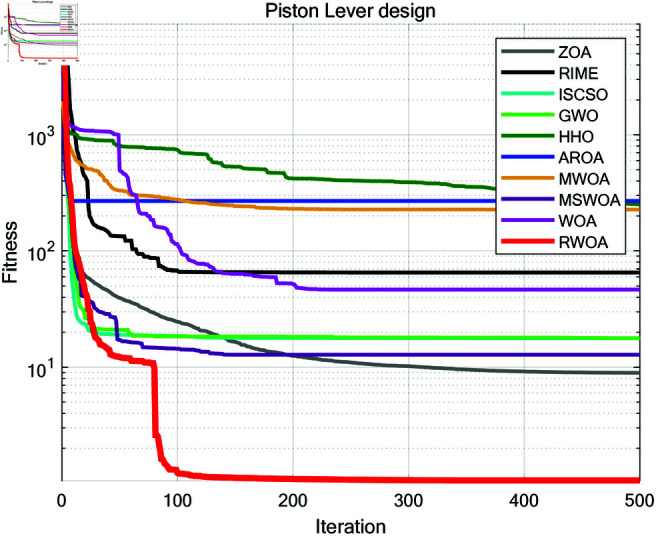
Iteration curves of the algorithms in piston lever design.

The experimental results are shown in Fig 26 and Table 10. As shown in Table 10, RWOA significantly outperformed the other algorithms in terms of both optimization accuracy and stability for the piston lever design problem. This demonstrates that RWOA had superior solving capabilities when handling this type of problem. This indicates that RWOA had a significant advantage in handling such optimization problems.

### 8.8 Reactor network

**Fig 27:**
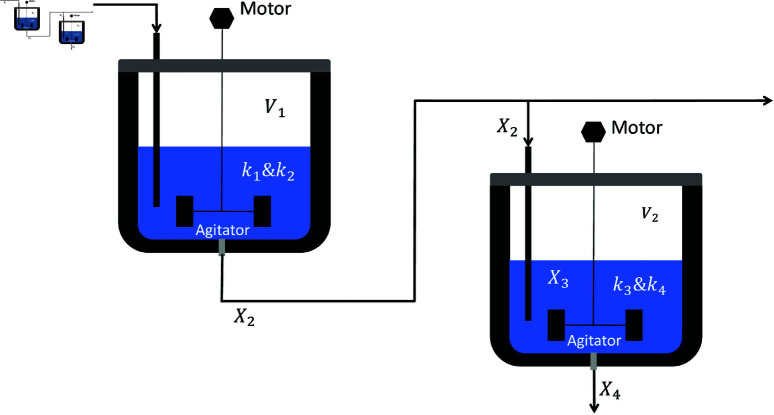
The structure of a reactor network.

Fig 27 is the structure of a reactor network. The reactor network design problem aims to optimize the configuration of chemical reactors in chemical plants, ordering to achieve a more efficient chemical reaction process. This involves selecting reactor types, configuring their arrangements, and allocating fluid flow rates, with the objective of maximizing product concentration. Optimization is achieved by adjusting the configuration and operating conditions of reactors to improve reaction efficiency, reduce energy consumption, or enhance product quality. The problem includes four variables representing the concentrations at different reaction stages: $x_{1}$ for the reactant concentration in the first reactor, $x_{2}$ for the product concentration in the first reactor, $x_{3}$ for the reactant concentration in the second reactor, and $x_{4}$ for the final product concentration. The constraints $g_{1}$ through $g_{4}$ are defined as follows: $g_{1}$ represents the balance of reactants and products in the first reactor, $g_{2}$ enforces mass conservation between the first and second reactors, $g_{3}$ maintains equilibrium for the reactant concentration between the reactors, and $g_{4}$ ensures mass conservation between intermediate and final products. The problem of reactor network design is modeled below.

*Variable:*


$$\begin{array}{lll} \begin{array}{cc} x & =[x_{1},x_{2},x_{3},x_{4},x_{5},x_{6}] \end{array} & \; & \end{array}$$

*Minimize:*


$$\begin{array}{ccc} y=x(4)+punishment & & \end{array}$$ (70)

*Subject to:*


$$\begin{array}{c} g(x)=\sqrt{x_{5}}+\sqrt{x_{6}}-4\leq 0 \end{array}$$ (71)

$$\begin{array}{c} h_{1}(x)=x_{1}+k_{1}\cdot x_{2}\cdot x_{5}-1=0; \end{array}$$ (72)

$$\begin{array}{c} h_{2}=x_{2}-x_{1}+k_{2}\cdot x_{2}\cdot x_{6}=0; \end{array}$$ (73)

$$\begin{array}{c} h_{3}(x)=x_{3}+x_{1}+k_{3}\cdot x_{3}\cdot x_{5}-1=0; \end{array}$$ (74)

$$\begin{array}{c} h_{4}=x_{4}-x_{3}+x_{2}-x_{1}+k_{4}\cdot x_{4}\cdot x_{6}=0; \end{array}$$ (75)

*Where:*


$$\begin{array}{lll} k_{1}=0.09755988;\;k_{2}=0.99\cdot k_{1};\;k_{3}=0.0391908;\;k_{4}=0.9\cdot k_{3}; & \; & \end{array}$$

$$\begin{array}{lll} \begin{array}{c} punishment=10^{3}\cdot \max{\left(0,g(x)\right)}^{2}+10^{3}\cdot \overset{4}{\underset{i=1}{\sum }}h_{i}{\left(x\right)}^{2} \end{array} & \; & \end{array}$$

*Variable range:*


$$\begin{array}{lll} 0.00001\leq x_{1}\leq 1;\;0.00001\leq x_{2}\leq 1;\;0.00001\leq x_{3}\leq 1;\;0.00001\leq x_{4}\leq 1; & \; & \end{array}$$

$$\begin{array}{lll} 0.00001\leq x_{5}\leq 16;\;0.00001\leq x_{6}\leq 16; & \; & \end{array}$$

**Fig 28:**
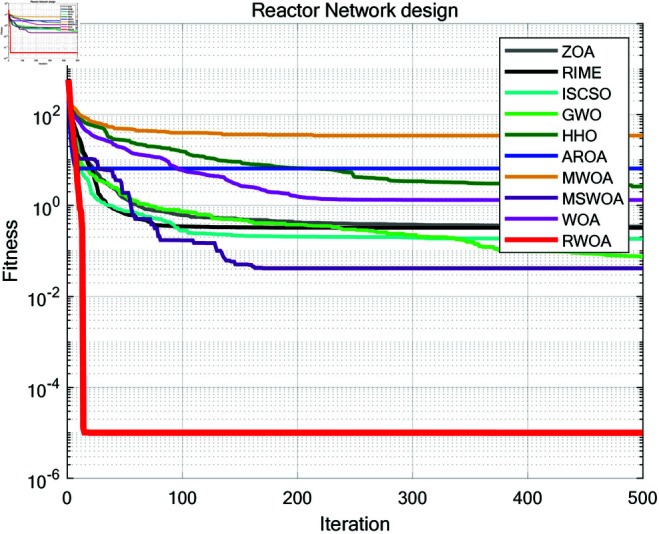
Iteration curves of the algorithms in reactor network design.

The experimental results are shown in Fig 28 and Table 10. As shown in Table 10, RWOA significantly outperformed the other algorithms in terms of both optimization accuracy and stability for the Reactor Network design problem. This demonstrates that RWOA had superior solving capabilities when handling this type of problem. This indicates that RWOA had a significant advantage in handling such optimization problems.

### 8.9 Gas transmission system

**Fig 29:**
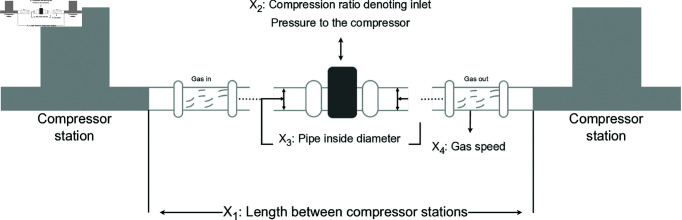
The structure of a gas transmission system.

The gas transmission system, as shown in Fig 29, is a crucial component of the modern energy supply chain, widely used in various industries, urban natural gas supply, and multinational energy transportation. Since the transportation of natural gas relies on Gas Transmission Compressors and pipeline networks, the design optimization of these devices is essential to ensuring energy transmission efficiency and reducing energy waste. The objective of the Gas Transmission Compressor optimization problem is to design and optimize the parameters of the natural gas transmission compressor, so that the compressor can deliver optimal performance under different working conditions, reduce energy consumption, extend service life, and minimize costs. The Gas Transmission Compressor optimization problem involves four design variables and one constraint. The meanings of the variables $x_{1}$ to $x_{4}$ are: $x_{1}$ indicates the length between compressor stations; $x_{2}$ indicates the compression ratio denoting inlet pressure to the compressor; $x_{3}$ indicates the pipe inside diameter; $x_{4}$ indicates the gas speed on the output side. The mathematical modeling of the Gas Transmission Compressor optimization problem is as follows:

*Variable:*


$$\begin{array}{lll} \begin{array}{cc} x & =[x_{1},x_{2},x_{3},x_{4}] \end{array} & \; & \end{array}$$

*Minimize:*


$$\begin{array}{ccc} f(x)=8.61\cdot 10^{5}x_{1}^{\frac{1}{2}}x_{2}x_{3}^{-\frac{2}{3}}x_{4}^{-\frac{1}{2}}+3.69\cdot 10^{4}x_{3}+7.72\cdot 10^{8}x_{1}^{-1}x_{2}^{0.219}-765.43\cdot 10^{6}x_{1}^{-1}+punishment & & \end{array}$$ (76)

*Subject to:*


$$\begin{array}{ccc} g(x)=x_{4}x_{2}^{-2}+x_{2}^{-2}-1\leq 0; & & \end{array}$$ (77)

*Where:*


$$\begin{array}{lll} k_{1}=0.09755988;\;k_{2}=0.99\cdot k_{1};\;k_{3}=0.0391908;\;k_{4}=0.9\cdot k_{3}; & \; & \end{array}$$

$$\begin{array}{lll} \begin{array}{c} punishment=10^{3}\cdot max{\left(0,g(x)\right)}^{2} \end{array} & \; & \end{array}$$

*Variable range:*


$$\begin{array}{lll} 20<x_{1}<50;\;1<x_{2}<10;\;20<x_{3}<45;\;0.1<x_{4}<60 & \; & \end{array}$$

**Fig 30:**
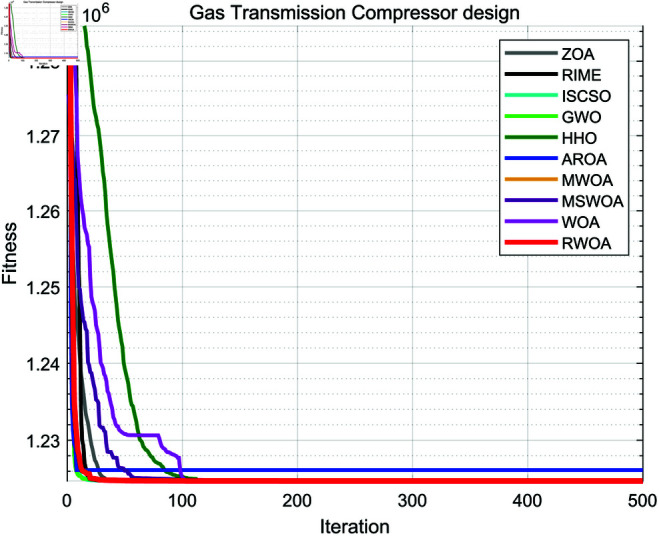
Iteration curves of the algorithms in gas transmission system design.

The experimental results are shown in Fig 30 and Table 10. As shown in Table 10, RWOA significantly outperformed the other algorithms in terms of both optimization accuracy and stability for the Gas Transmission System design problem. This demonstrates that RWOA had superior solving capabilities when handling this type of problem. This indicates that RWOA had a significant advantage in handling such optimization problems.

## 9 Conclusion and future work

RWOA introduced the Goode Nodes Set Method to generate uniformly distributed populations. It incorporated newly designed strategies, including the Hybrid Collaborative Exploration Strategy, Spiral Encircling Prey Strategy, Enhanced Spiral Updating Strategy, and Enhanced Cauchy Mutation based on Differential Evolution. RWOA also incorporated a newly designed update method for parameter *a*, which helped balance global exploration and local exploitation.

To validate the effectiveness of the RWOA improvements, four experiments were conducted:

1. We systematically removed six of the improvements in RWOA and compared the results with the complete RWOA to evaluate the effectiveness of each individual improvement strategy;
2. A Parameter Sensitivity Analysis experiment was conducted to select the most suitable scaling factor *β* for RWOA;
3. A Qualitative Analysis experiment was performed, and the results showed that RWOA effectively balanced exploration and exploitation during optimization, achieving high convergence accuracy, fast convergence speed, and maintaining population diversity;
4. RWOA was compared with state-of-the-art (SOTA) metaheuristic algorithms on 23 classical benchmark functions to verify its superiority.

Furthermore, RWOA was applied to nine engineering design optimization problems, demonstrating its feasibility in real-world engineering design optimization. RWOA provided a novel approach for the application of WOA in engineering design.

RWOA effectively addressed the shortcomings of the classic WOA, such as premature convergence, low population diversity in later iterations, slow convergence speed, low convergence accuracy, and the imbalance between exploration and exploitation. Compared with other metaheuristic algorithms, RWOA shown strong competitiveness. Although RWOA provided competitive results for numerical optimization and engineering design tasks, its time consumption was comparable to that of WOA in common numerical optimization problems, primarily due to the introduction of Enhanced Cauchy Mutation based on Differential Evolution. However, in large-scale complex numerical optimizations, the Enhanced Cauchy Mutation based on Differential Evolution led to increased computational time for RWOA. Therefore, RWOA was not suitable for solving large-scale real-time problems.

Future work will involve rigorous testing on manufacturing prototypes, validation in real-world scenarios, and consideration of real-world constraints to enhance the reliability and effectiveness of the optimization process, aiming to achieve more reliable and efficient mechanical designs. We recommend RWOA as a tool for design, simulation, and manufacturing, meeting the needs of contemporary industry. Furthermore, future research will focus on further exploring RWOA’s applications in APS, data clustering, path planning, and neural network parameter optimization.

## Appendix A. Details of the benchmark fucntions

To support the experimental study in this paper, we used the Standard Benchmark Functions. The relevant data has been uploaded to Figshare, and the link for the specific modeling of Standard Benchmark Functions is: https://figshare.com/account/items/28440863/edit, for reference and further analysis by the readers.
